# Neuronal hyperexcitability drives central and peripheral nervous system tumor progression in models of neurofibromatosis-1

**DOI:** 10.1038/s41467-022-30466-6

**Published:** 2022-05-19

**Authors:** Corina Anastasaki, Juan Mo, Ji-Kang Chen, Jit Chatterjee, Yuan Pan, Suzanne M. Scheaffer, Olivia Cobb, Michelle Monje, Lu Q. Le, David H. Gutmann

**Affiliations:** 1 Department of Neurology, Washington University School of Medicine, St. Louis, MO 63110 USA; 2 Department of Dermatology, University of Texas, Southwestern, Dallas, TX 75390 USA; 3 Department of Neurology and Neurological Sciences, Stanford University, Stanford, CA 94305 USA; 4 Howard Hughes Medical Institute, Stanford University, Stanford, CA 94305 USA

**Keywords:** CNS cancer, Cancer in the nervous system, Cancer models

## Abstract

Neuronal activity is emerging as a driver of central and peripheral nervous system cancers. Here, we examined neuronal physiology in mouse models of the tumor predisposition syndrome Neurofibromatosis-1 (NF1), with different propensities to develop nervous system cancers. We show that central and peripheral nervous system neurons from mice with tumor-causing *Nf1* gene mutations exhibit hyperexcitability and increased secretion of activity-dependent tumor-promoting paracrine factors. We discovered a neurofibroma mitogen (COL1A2) produced by peripheral neurons in an activity-regulated manner, which increases *NF1*-deficient Schwann cell proliferation, establishing that neurofibromas are regulated by neuronal activity. In contrast, mice with the Arg1809Cys *Nf1* mutation, found in NF1 patients lacking neurofibromas or optic gliomas, do not exhibit neuronal hyperexcitability or develop these NF1-associated tumors. The hyperexcitability of tumor-prone *Nf1*-mutant neurons results from reduced *NF1*-regulated hyperpolarization-activated cyclic nucleotide-gated (HCN) channel function, such that neuronal excitability, activity-regulated paracrine factor production, and tumor progression are attenuated by HCN channel activation. Collectively, these findings reveal that *NF1* mutations act at the level of neurons to modify tumor predisposition by increasing neuronal excitability and activity-regulated paracrine factor production.

## Abstract (web summary)

Neuronal activity is emerging as a driver of nervous system tumors. Here, the authors show in mouse models of Neurofibromatosis-1 (NF1) that *Nf1* mutations differentially drive both central and peripheral nervous system tumor growth in mice through reduced hyperpolarization-activated cyclic nucleotide-gated (HCN) channel function.

## Introduction

While the acquisition of genetic or epigenetic aberrations in preneoplastic cells is an obligate event in tumor formation and progression, cancer growth is also dictated by paracrine factors produced by non-neoplastic cells in the local tumor microenvironment^1–4^. These tumor–stroma interactions are nicely illustrated in the setting of the neurofibromatosis-1 (NF1) genetic cancer predisposition syndrome. Patients with NF1, born with a germline mutation in the *NF1* tumor suppressor gene, are prone to developing various tumors, including central and peripheral nervous system tumors, as well as malignancies of the adrenal glands, muscle, blood, and breast^5^. In NF1 nervous system tumors, paracrine factors from T cells, monocytes (macrophages, microglia), and mast cells generate a supportive tumor microenvironment necessary for continued peripheral (neurofibromas) and central (gliomas) nervous system tumor expansion^6–11^. As such, plexiform neurofibroma (pNF) formation and growth is controlled by the interplay of mast cells, macrophages, leukocytes, and fibroblasts through paracrine factor elaboration^10–16^, whereas T cells^7,17,18^ and microglia^6,19–21^ influence glioma growth through cytokine (Ccl4, Ccl5) signaling.

In addition to the critical contributions from immune system cells, we have recently shown that *NF1* mutation in neurons synergizes with light-induced retinal ganglion cell activity to regulate neuroligin-3 (NLGN3) shedding and *Nf1*-optic pathway glioma (*Nf1-*OPG) initiation and growth^22^. This finding builds upon prior reports establishing that neurons and neuronal activity increase high-grade glioma growth through the secretion of paracrine factors, like NLGN3 and brain-derived neurotrophic factor (BDNF), in an activity-dependent manner^23,24^ or by forming bona fide AMPA receptor-dependent neuron-to-glioma synapses^25,26^. Moreover, these effects of neuronal activity on high-grade glioma growth are amplified by glioma-induced hyperexcitability of neurons^26–31^.

To further elucidate the contribution of neuronal activity to central and peripheral nervous system tumor development, we focused on NF1, where affected individuals are prone to developing tumors intimately associated with nerves, including OPGs and pNFs^32–34^. Using these preclinical models, we previously demonstrated that different germline *Nf1* mutations have dramatically different effects on plexiform neurofibroma and OPG formation in mice^17,35,36^, suggesting that the specific *NF1* germline mutation may regulate tumorigenesis at the level of non-neoplastic cells.

In this study, we leveraged a common, naturally occurring *NF1* missense mutation (c.5425C > T; p.Arg1809Cys) found in patients with NF1 who do not develop OPGs or neurofibromas. Exploiting this unique mutation, we employed a combination of human-induced pluripotent stem cell (hiPSC) and *Nf1*-mutant mouse lines to demonstrate that central (retinal ganglion cells; RGCs) and peripheral (sensory neurons and dorsal root ganglion cells; DRGs) nervous system neurons support tumor growth by secreting paracrine factors necessary for tumor progression in an *Nf1* mutation- and neuronal activity-dependent manner. In contrast to mice with other NF1 patient germline *NF1* gene mutations, mice with the Arg1809Cys mutation, like NF1 patients with this mutation, do not form pNFs or OPGs and their DRGs and RGCs, respectively, do not exhibit the RAS-independent neuronal hyperexcitability seen in tumor-forming *Nf1*-mutant central and peripheral nervous system neurons. Based on prior studies revealing that the *NF1* protein, neurofibromin, binds to and regulates hyperpolarization-activated cyclic nucleotide-gated (HCN) channels^37^ and that HCN channels directly modulate neuronal excitability^38,39^, we now show that HCN channel dysregulation is responsible for *Nf1*-mutant central and peripheral nervous system neuronal hyperexcitability and consequently increased tumor-driving paracrine factor release, such that HCN channel targeting (using the anti-seizure medication lamotrigine) blocked *Nf1*-OPG progression in vivo. Moreover, we demonstrate that tumor-causing *Nf1* mutations in neurons regulate neuronal production of paracrine factors through both visual experience (light)-evoked neuronal activity, as well as HCN channel dysregulation-mediated baseline neuronal hyperexcitability, highlighting the essential role of neuronal activity in NF1-associated nervous system tumor progression.

## Results

### Arg1809Cys *Nf1*-conditional mutant mice do not develop optic pathway gliomas

The NF1 patient c.5425C > T p.Arg1809Cys *NF1* mutation^40^ was engineered in mice on a C57Bl/6J background by CRISPR/Cas9 targeting and confirmed by direct sequencing. Wild-type (WT) and heterozygous Arg1809Cys *Nf1-*mutant mice (*Nf1*^+/1809^) were born from heterozygous *Nf1*^*+/1809*^ parents with the expected Mendelian ratios (Supplementary Fig. 1A). However, no homozygous *Nf1*^1809/1809^ mice were born, suggesting embryonic lethality, as seen with conventional *Nf1* knockout mice^41,42^. Heterozygous mice had similar weights as WT littermate controls and two genetically engineered mouse (GEM) strains harboring different germline NF1 patient-derived germline *Nf1* gene mutations (c.2041C > T, p.R681X^35,36^; c.3827G > C, p.R1276P^43^) (Supplementary Fig. 1B).

**Fig. 1:**
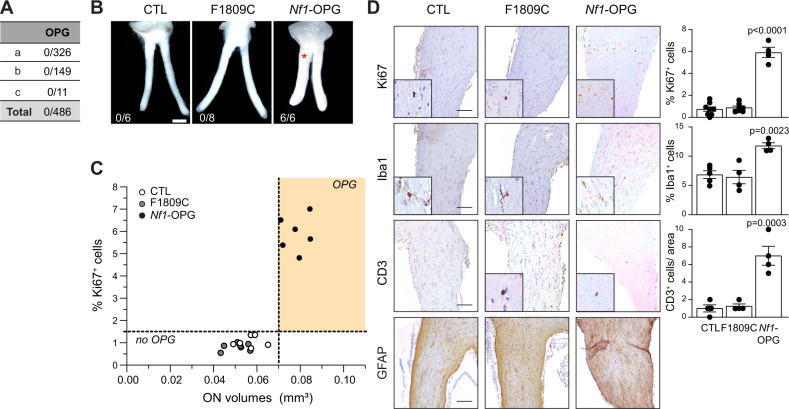
Arg1809Cys *Nf1-*mutant mice do not develop optic gliomas following somatic *Nf1* inactivation. **A** Incidence of optic pathway glioma (OPG) in NF1 patients harboring the c.5425C > T *NF1* germline mutation. (a)^44^, (b)^45^, (c)^40^. **B** Representative images of dissected optic nerves from control (*Nf1*^f/f^; CTL) and *Nf1-*mutant mice harboring conditional somatic *Nf1* inactivation in neuroglial progenitors (*Nf1*^f/1809^; *GFAP-*Cre, F1809C; *Nf1*^f/neo^; *GFAP*-Cre, *Nf1*-OPG). Whereas *Nf1*-OPG mice form OPGs (red asterisk), CTL and F1809C mice do not. The number of mice that formed OPGs is shown in each panel. Scale bar: 1 mm. **C** Graph demonstrating the relationship between optic nerve volumes and Ki67^+^ cells in CTL, F1809C, and *Nf1*-OPG optic nerves. *n* = 6 for all groups. **D** Ki67, Iba1, CD3, and GFAP immunostaining of optic nerves in CTL, F1809C, and *Nf1*-OPG mice. Scale bars, 50 µm. (Ki67: CTL *n* = 8, F1809C *n* = 7, *Nf1*-OPG *n* = 4, *P* < 0.0001; Iba1: CTL *n* = 5, F1809C *n* = 4, *Nf1*-OPG *n* = 4, *P* = 0.0023; CD3: CTL *n* = 4, F1809C *n* = 4, *Nf1*-OPG *n* = 4, *P* = 0.0003). Data are represented as means ± SEM. One-way ANOVA with Dunnett’s post-test correction. *P* values are indicated within each panel. ns, not significant. Source data are provided as a Source Data file.

Like patients with the R1809C germline *NF1* gene mutation who lack OPGs (Fig. 1A)^40,44,45^, mice harboring a germline *Nf1*^R1809C^ mutation with somatic loss of *Nf1* in neuroglial progenitor cells, the optic glioma initiating cells (*Nf1*^f/1809^; hGFAP-Cre mice (F1809C)), did not develop OPGs at 3 months of age (0/8; Fig. 1B). In contrast, all *Nf1*^f/neo^; hGFAP-Cre mice (*Nf1*-OPG), where the germline *Nf1* inactivation results from the insertion of a neomycin cassette into exon 31 of the *Nf1* gene^41,42^, developed OPGs (6/6) with increased optic nerve volumes (0.079 mm^3^ Fig. 1C), proliferative indices (5.9% Ki67^+^ cells), microglia (11.8% Iba1^+^ cells), T cells (7 CD3^+^ cells) and GFAP^+^ cells, as previously reported^46^ (Fig. 1D). Importantly, optic nerves from *Nf1*^f/1809^; hGFAP-Cre mice were indistinguishable from *Nf1*^f/f^ controls (CTL) with respect to optic nerve volume (*Nf1*^f/1809^; hGFAP-Cre, 0.05 mm^3^ CTL, 0.057 mm^3^), proliferative index (*Nf1*^f/1809^; hGFAP-Cre, 0.8%; CTL, 1.03 Ki67^+^ cells), microglia content (*Nf1*^f/1809^; hGFAP-Cre, 6.4%; CTL, 6.8% Iba1^+^ cells), T-cell content (*Nf1*^f/1809^; hGFAP-Cre, 1.2; CTL, 1 CD3^+^ cells) and GFAP immunoreactivity (Fig. 1C, D). Taken together, these findings demonstrate that mice with the Arg1809Cys germline *Nf1* mutation, like their human counterparts, do not develop OPGs.

### OPG-associated *Nf1*-mutant CNS neurons are hyperexcitable

**Fig. 2:**
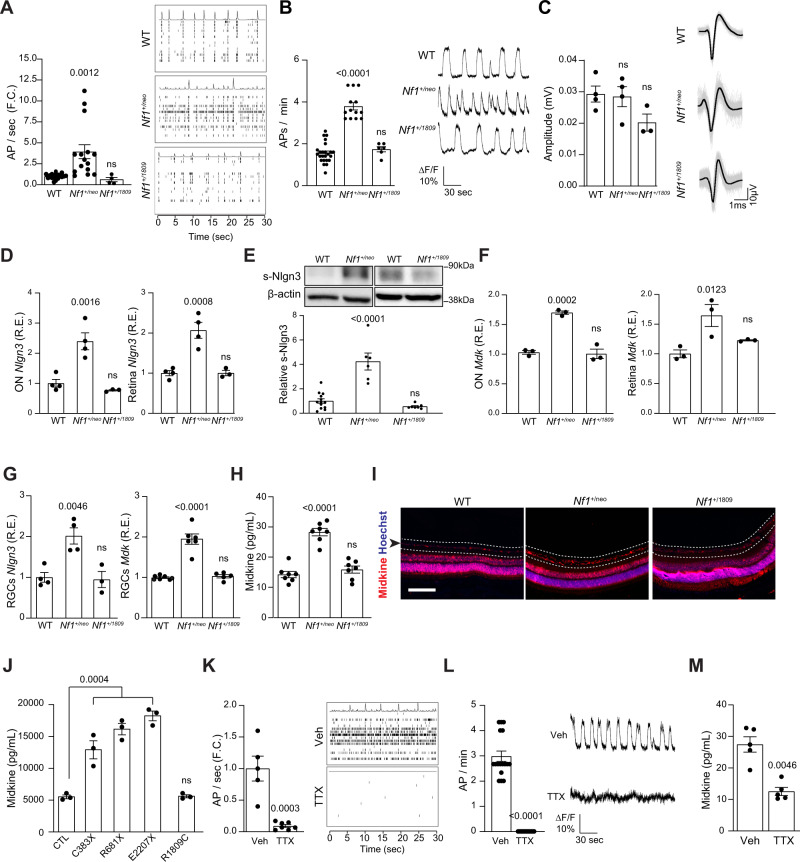
OPG-associated *Nf1-*mutant neurons have increased activity and OPG-promoting factor production. *Nf1*^+/neo^, but not *Nf1*^+/1809^, RGC neuron activity (AP firing rates), as measured by (**A**) multi-electrode arrays (CTL *n* = 27, *Nf1*^+/neo^ *n* = 15, *P* = 0.0012, *Nf1*^+/1809^ *n* = 4), or (**B**) calcium imaging (CTL *n* = 24, *Nf1*^+/neo^ *n* = 13, *P* < 0.0001, *Nf1*^+/1809^ *n* = 6), is elevated relative to WT RGC neurons. Each dot represents (**A**) the averge of a minimum of three technical replicates for a single animal, or (**B**) a single neuron. Right panels depict representative (**A**) spike plots of entire multi-electrode array well recordings over 30 s and (**B**) traces of neuronal activity represented as fluorescence differentials over 3 min. **C** The amplitudes of action potentials are similar in *Nf1*^*+/neo*^ and *Nf1*^*+/1809*^ RGC neurons relative to WT controls (CTL *n* = 4, *Nf1*^+/neo^ *n* = 4, *Nf1*^+/1809^ *n* = 3). ns not significant. Right panels: representative traces of action potentials recorded over 3 ms (gray). The average of the action potentials is shown in black. **D** Neuroligin-3 transcript (*Nlgn3)* relative expression (CTL *n* = 4, *Nf1*^+/neo^ *n* = 4, O.N. *P* = 0.0016, retina *P* = 0.0008, *Nf1*^+/1809^ *n* = 3, ns), and (**E**) soluble neuroligin-3 (s-Nlgn3; CTL *n* = 13, *Nf1*^+/neo^ *n* = 6, *P* < 0.0001*, Nf1*^+/1809^ *n* = 7, ns) are increased in *Nf1*^+/neo^ optic nerves (ON) and retinae relative to WT and *Nf1*^+/1809^ counterparts. β-actin was used as a loading control. **F** Midkine transcript (*Mdk)* relative expression is increased in whole optic nerves and retinae from *Nf1*^+/neo^ mice relative to WT controls and *Nf1*^+/1809^ mice. *n* = 3 for all groups. O.N. *Mdk* R.E., *P* = 0.0002; retinal *Mdk* R.E., *P* = 0.0123. **G** *Nlgn3* (CTL *n* = 4, *Nf1*^+/neo^ *n* = 4, *Nf1*^+/1809^ *n* = 3; *P* = 0.0046) and *Mdk* (CTL *n* = 7, *Nf1*^+/neo^ *n* = 6, *Nf1*^+/1809^ *n* = 5; *P* < 0.0001) transcript relative expression is increased in *Nf1*^*+/neo*^ retinal ganglion cell (RGC) neurons relative to WT and *Nf1*^+/1809^ RGCs. **H**, **I** Midkine protein expression is elevated in (**H**) the *Nf1*^+/neo^ conditioned media (CM) from RGCs in vitro (*n* = 7 for all groups; *P* < 0.0001), and (**I**) the RGC layer of *Nf1*^+/neo^ mice relative to WT and *Nf1*^+/1809^ mice (*n* = 5 for each group). Scale bar, 50 µm. Dotted lines and arrow highlight the RGC layer. **J** Midkine expression is elevated in human CNS excitatory *NF1*^C383X^ (*P* = 0.0004), *NF1*^R681X^ (*P* < 0.0001) and *NF1*^E2207X^ (*P* < 0.0001) mutant neurons, but not *NF1*^R1809C^ neurons, relative to controls (CTL). *n* = 3 for all groups. **K**, **L** Tetrodotoxin (TTX; 1 µM) reduced the AP firing rate of *Nf1*^+/neo^ RGC neurons relative to controls, as measured by (**K**) multi-electrode arrays (vehicle *n* = 5, TTX *n* = 7; *P* = 0.0003) and (**L**) calcium imaging (vehicle *n* = 17, TTX *n* = 17; *P* < 0.0001). Right panels: representative (**K**) spike plots of entire multi-electrode array well recordings over 30 s, and (**L**) traces of neuronal activity over 3 min. **M** TTX reduced midkine secretion by *Nf1*^+/neo^ RGC neurons. *n* = 5 for all groups (*P* = 0.0046). Data are represented as means ± SEM. **B**–**H**, **J** One-way ANOVA with Dunnett’s post-test correction, or (**A**, **K**–**L**) two-tailed unpaired and (**M**) two-tailed paired Student’s *t* test. *P* values are indicated within each panel. ns, not significant. Source data are provided as a Source Data file.

Prior studies from our laboratories have shown that OPG growth in *Nf1-*mutant mice (*Nf1*^f/neo^; hGFAP-Cre) is initiated by neuronal activity-dependent paracrine signaling^22^. In these mice, neuroligin-3 (Nlgn3) is shed in the *Nf1*-mutant (*Nf1*^*+/neo*^) optic nerve in an activity-dependent manner, such that genetic or pharmacological blockade of Nlgn3 shedding inhibits glioma initiation and progression^22^. Based on these findings, we first examined the neuronal activity of primary WT, *Nf1*^+/neo^, and *Nf1*^+/1809^ RGCs using multi-electrode arrays (Fig. 2A) or calcium imaging (Fig. 2B) after 10 days in vitro. We found that the *Nf1*^+/neo^, but not the *Nf1*^+/1809^, neurons had increased activity relative to WT RGCs, as measured by action potential (AP) firing rates (2.5–3.9-fold increase relative to WT control; Fig. 2A, B). No change in neuronal action potential amplitudes were noted in *Nf1*^+/neo^ or *Nf1*^+/1809^ neurons relative to WT controls (Fig. 2C). This suggests that *Nf1* mutations associated with tumor formation cause RGC neurons to be hyperexcitable.

### OPG-associated *Nf1*-mutant CNS neurons secrete tumor-promoting factors in an activity-dependent manner

To determine whether increased RGC activity triggers the secretion of the two known neuronal OPG-promoting factors, Nlgn3 and midkine^7,22^, we assessed their transcript and protein expression levels both in vitro and in vivo. Optic nerves (ONs), RGCs (Supplementary Fig. 2A), and RGCs within the intact retinae from *Nf1*^+/neo^, but not *Nf1*^+/1809^, mice had increased expression of *Nlgn3* RNA (2.0–2.3-fold increase; Fig. 2D, G), soluble cleaved Nlgn3 protein (s-Nlgn3; Fig. 2E), *Mdk* RNA (1.6-fold increase; Fig. 2F) and midkine protein (2.2-fold increase; Fig. 2H, I) expression relative to WT controls. Increased midkine expression was also detected in RGCs from *Nf1*^+/R681X^-mutant mice (Supplementary Fig. 2B), another mouse strain that develops optic gliomas following somatic *Nf1* inactivation in neuroglial progenitors, as well as in *Nf1*^+/neo^, but not *Nf1*^+/1809^, mouse primary hippocampal neurons (Supplementary Figs. 2A and 3A, B). In addition, hippocampal neurons from *Nf1*^+/neo^ mice similarly exhibited hyperexcitability (Supplementary Fig. 3C).

The correlation between neuronal midkine production and tumor risk is reinforced in human iPSC-derived central nervous system neurons (Supplementary Fig. 2C). Midkine expression is increased both in excitatory (Fig. 2J) and inhibitory (Supplementary Fig. 2D) neurons harboring *NF1* mutations that are found in NF1 patients that develop OPGs (c.1149 C  >  A, p.Cys381X; c.2041 C  >  T, pArg681X; c.6619 C  >  T, p.Gln2207X)^47^, but not in *NF1*^+/R1809C^ neurons, relative to controls (CTL). Similarly, *Adam10* transcript expression was only increased in *Nf1*^+/neo^, but not in *Nf1*^+/1809^, mouse retinae, ONs, and RGCs (Supplementary Fig. 2F–H). In contrast, neither *Nf1*^+/neo^ nor *Nf1*^+/1809^ PNS (DRG) sensory neurons had increased *Nlgn3* (Supplementary Fig. 2I) or midkine (Supplementary Fig. 2J, K) expression relative to WT controls, highlighting the selective upregulation of Nlgn3 and midkine in CNS, rather than in PNS, neurons.

As part of a neuron-immune-cancer cell axis in *Nf1*-OPG, *Nf1*-mutant neurons secrete midkine to induce T-cell Ccl4 expression, which in turn, results in microglial elaboration of Ccl5, an obligate OPG growth factor^7,18,19^. To ascertain whether this molecular circuitry is intact in mice harboring the *Nf1*^+/1809^ mutation, and to exclude defects in other stromal cells (T cells and microglia) that might be additionally responsible for the observed lack of optic gliomas in *Nf1*^f/1809^; hGFAP-Cre mice, we examined the ability of *Nf1*^+/1809^ T cells and microglia to secrete Ccl4 in response to midkine and Ccl5 in response to Ccl4, respectively (Supplementary Fig. 2L, M). Both *Nf1*^+/1809^ T cells and microglia responded to midkine and Ccl4, respectively, similar to their *Nf1*^+/neo^ counterparts^7^. Therefore, the lack of OPG formation likely reflects the failure of *Nf1*^+/1809^ neurons to produce glioma-promoting trophic factors. Importantly, blockade of *Nf1*^+/neo^ neuronal activity with 1 µM tetrodotoxin (TTX) ( > 80-fold decrease; Fig. 2K, L) reduced midkine levels (1.9-fold decrease; Fig. 2M), similar to TTX effects on Nlgn3^22^, confirming that both Nlgn3 and midkine secretion are neuronal activity-dependent and reversible by pharmacological treatment.

### HCN channel activity regulates midkine production in OPG-associated *Nf1* RGCs

**Fig. 3:**
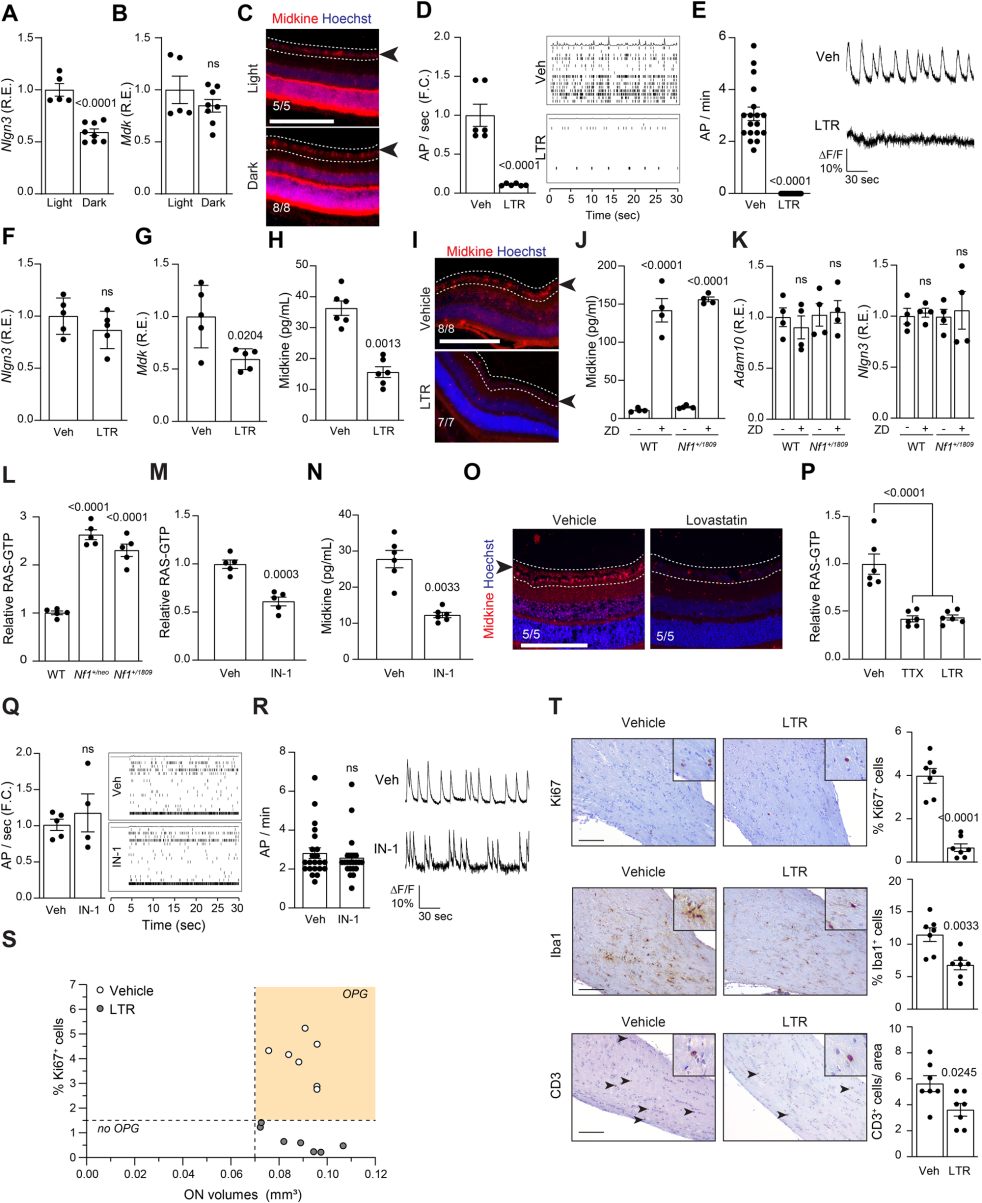
OPG-associated *Nf1-*mutant neuronal hyperexcitability is HCN channel-dependent. **A** Neuroligin (*Nlgn3;* *P* < 0.0001) but not (**B**) midkine (*Mdk*; ns not significant) transcript relative expression is decreased in retinae of *Nf1*^+/neo^ mice following dark-rearing from 4 to 8 weeks. Light-reared *n* = 5, dark-reared *n* = 8. **C** Midkine expression is not reduced in the RGC layer (dotted lines, black arrow) or retinae in 8-week-old *Nf1*^+/neo^ mice following dark-rearing from 4 to 8 weeks. Light-reared *n* = 5, dark-reared *n* = 8. **D**, **E** RGC activity is reduced following 200 µM lamotrigine (LTR) treatment, as measured by (**D**) multi-electrode array (vehicle *n* = 6; LTR *n* = 6; *P* < 0.0001), or (**E**) calcium imaging (vehicle *n* = 18; LTR *n* = 18; *P* < 0.0001). Right panels: representative (**D**) spike plots of entire multi-electrode array well recordings over 30 s, and (**E**) traces of neuronal activity over 3 min. **F** *Nlgn3* relative expression is unaltered (ns not significant), while (**G**) *Mdk* transcript relative expression is decreased in retinae of *Nf1*^+/neo^ mice following LTR treatment in vivo. *n* = 5 for all groups. *P* = 0.0204. **H**, **I** Midkine expression is reduced in (**H**) *Nf1*^+/neo^ RGC neurons in vitro (*n* = 6 for all groups; *P* = 0.0013), and (**I**) in the RGC layer (dotted lines, black arrow) of retinae in 12-week-old *Nf1*^f/neo^; GFAP-Cre (*Nf1*-OPG) mice following LTR treatment in vivo (vehicle *n* = 8; LTR *n* = 7). **J**, **K** ZD7288 (ZD) treatment (30 µM) of WT and *Nf1*^+/1809^ RGC neurons (**J**) increased midkine production (*P* < 0.0001), but (**K**) did not alter *Adam10* or *Nlgn3* transcript expression in vitro (ns, not significant). *n* = 4 for all groups. **L**, **M** RAS activity is elevated in *Nf1*^+/neo^ and *Nf1*^+/1809^ (**L**) RGC neurons relative to WT controls (*P* < 0.0001), and (**M**) is reduced in *Nf1*^+/neo^ neurons following IN-1 treatment (1 µM; *P* = 0.0003). *n* = 5 for all groups. **N** Midkine levels are reduced in *Nf1*^+/neo^ RGC neurons following IN-1 treatment. *n* = 6 for all groups; *P* = 0.0033. **O** RGC layer (dotted lines, black arrow) midkine expression is reduced following lovastatin treatment of 12-week-old *Nf1*-OPG animals in vivo. *n* = 5 for all groups. **P** RAS-GTP is reduced in TTX (1 µM)- and LTR-treated *Nf1*^+/neo^ RGCs. *n* = 6 for all groups, *P* < 0.0001. **Q**, **R** *Nf1*^+/neo^ RGC neuron AP firing rate is not reduced following IN-1 treatment, as measured by (**Q**) multi-electrode array (vehicle *n* = 5; IN-1 *n* = 4), or (**R**) calcium-imaging recordings (vehicle *n* = 22; IN-1 *n* = 22). Right: **Q** spike plots of entire multi-electrode array well recordings over 30 s, and (**R**) traces of neuronal activity over 3 min. ns, not significant. **S** Graph demonstrating the relationship between optic nerve volumes and Ki67^+^ cells in vehicle- and LTR-treated *Nf1*-OPG optic nerves. *n* = 7 for both groups. **T** LTR-treated *Nf1*-OPG mouse optic nerves have reduced Ki67^+^ (*P* < 0.0001), Iba1^+^ (*P* = 0.0033) and CD3^+^ cells (*P* = 0.0245) relative to vehicle-treated *Nf1*-OPG mice. *n* = 7 for all groups. Scale bars, 100 µm. Data are represented as means ± SEM, (**A**, **B**, **D**–**G**, **M**, **Q**, **R**, **T**) unpaired two-tailed Student’s *t* test, (**H**, **N**) paired Student’s *t* test, (**J**–**L**, **P**) One-way ANOVA with (**J**) Tukey’s or (**K**, **L**, **P**) Dunnett’s post-test correction. *P* values are indicated within each panel. ns, not significant. Source data are provided as a Source Data file.

To determine whether light-induced retinal ganglion cell neuronal activity regulates midkine secretion in the optic nerve, *Nf1*^+/neo^ mice were reared either in 12 h light/dark cycles or completely in the dark for 4 weeks starting at 4 weeks of age. The retinae of dark-reared animals had decreased levels of *Nlgn3* (48% decrease; Fig. 3A) relative to light/dark-reared controls. In stark contrast, retinal *Mdk* RNA and protein expression were not affected by the decrease in visual experience (Fig. 3B, C), suggesting an alternative mechanism for neuronal activity-dependent midkine production.

Based on prior experiments demonstrating that HCN channels control neuronal hyperexcitability and that the *Nf1* mutation regulates HCN channel function^37^, we examined the effect of *Nf1* mutation on HCN channel function and neuronal excitability. *Hcn1* and *Hcn2* account for the majority of retinal *Hcn* channel expression; however, *Nf1* mutation (*Nf1*^+/neo^) does not alter *Hcn* levels (Supplementary Fig. 2N). To ascertain whether HCN channel function was responsible for the increased neuronal activity and Nlgn3/midkine production, we treated *Nf1*^+/neo^ RGC neurons with 200 µM lamotrigine (LTR), an HCN channel agonist, and assayed neuron activity for 3 min (Fig. 3D, E). Lamotrigine reduced the firing rates in *Nf1*^+/neo^ RGC neurons ( > 80% decrease; Fig. 3D, E). In striking contrast, while lamotrigine treatment of either heterozygous *Nf1*^+/neo^ or OPG-bearing *Nf1*^f/neo^; hGFAP-Cre mice in vivo did not change *Nlgn3* or *Adam10* RNA expression (Fig. 3F and Supplementary Fig. 2O–R), *Mdk* RNA (Fig. 3G) and protein levels were reduced in *Nf1*^+/neo^ retinae (1.9–2.3-fold decrease; Fig. 3H, I and Supplementary Fig. 2Q), optic nerves (1.7–2-fold decrease; Supplementary Figs. 2P and 3R) and RGCs (2.2-fold decrease; Fig. 3H) relative to vehicle-treated controls. Conversely, treatment of WT and *Nf1*^+/1809^ neurons with 30 µM of the HCN channel antagonist ZD7288 (ZD) resulted in a 14–15-fold increase in RGC neuron midkine production (Fig. 3J) but did not alter *Nlgn3* or *Adam10* RNA expression (Fig. 3K). Identical results were obtained using hippocampal neurons (Supplementary Fig. 3A–H), supporting the idea that baseline neuronal hyperexcitability mediated by HCN function is a shared feature of *Nf1*-mutant CNS neurons. As a complementary genetic approach, we infected wild-type neurons using three separate short hairpins against *Hcn1* and *Hcn2*. Both alone and in combination, infection of RGC and DRG neurons with the sh*Hcn1/2* constructs resulted in rapid neuronal death within 6 hours (Supplementary Fig. 4A, B), demonstrating that *Hcn1* and *Hcn2* presence is required for neuronal survival. Similarly, incubation of neurons with TTX, a drug that abolishes neuronal activity, also induces neuronal death within 6 hours(Supplementary Fig. 4C). Together, these data reveal the existence of an HCN channel-dependent mechanism for *Nf1*-mutant CNS tumor-associated neuronal midkine production.

### Increased *Nf1*-mutant neuron activity is not RAS-dependent

As the *NF1* protein (neurofibromin) functions a negative regulator of RAS activity (RAS-GTPase-activating protein), RAS-GTP levels were increased by 2.3–2.7-fold in *Nf1*^+/1809^ RGC and hippocampal neurons relative to WT controls, similar to *Nf1*^+/neo^ neurons (Fig. 3L and Supplementary Fig. 3I) and other mouse strains harboring NF1 patient-specific *Nf1* germline mutations^47^. The finding of similarly increased RAS-GTP in *Nf1*^+/1809^ CNS neurons suggests that RAS deregulation is not responsible for the failure of *Nf1*^f/1809^; hGFAP-Cre mice to form tumors. However, it does not exclude RAS as a potential signaling effector downstream of HCN channel activity. In this respect, treatment of *Nf1*^+/neo^ neurons with the pan-RAS inhibitor, IN-1, reduced RAS-GTP levels (Fig. 3M and Supplementary Fig. 3I), as well as midkine expression (Fig. 3N and Supplementary Fig. 3J). In addition, systemic treatment of *Nf1*^+/neo^; hGFAP-Cre mice with the RAS inhibitor lovastatin decreased RGC midkine expression in vivo (Fig. 3O), indicating that RAS operates to control midkine expression. Conversely, whereas inhibition of *Nf1*^+/neo^ neuronal activity by TTX and lamotrigine reduced RAS hyperactivation (Fig. 3P and Supplementary Fig. 3K), RAS (IN-1) inhibition had no effect on neuronal activity (Fig. 3Q, R and Supplementary Fig. 3L). Taken together, these results position RAS-mediated neuron midkine production downstream of HCN channel activity, and demonstrate that increased baseline excitability of tumor-associated *Nf1*-mutant neurons is RAS-independent.

### Increased HCN channel activity prevents OPG progression in vivo

To determine whether HCN channel function is critical for OPG formation, *Nf1*^f/neo^; hGFAP-Cre (*Nf1*-OPG) mice received intraperitoneal injections of lamotrigine from 6 to 8 weeks of age, at the time of early tumor evolution. Consistent with neuronal activity mediating *Nf1*-OPG progression, HCN activation by lamotrigine reduced OPG development at 3 months of age. Lamotrigine treatment did not decrease optic nerve volumes (1.5-fold increased volumes relative to WT controls; Fig. 3S), unlike dark-reared *Nf1*-OPG mice or those genetically lacking *Ngln3*^22^, where tumor initiation was completely prohibited. However, lamotrigine treatment resulted in reduced optic nerve proliferation (%Ki67^+^ cells; 5.7-fold decrease), as well as microglia (%Iba1^+^ cells; 1.7-fold decrease) and T-cell (CD3^+^ cells; 1.6-fold decrease) content, relative to vehicle-treated *Nf1*-OPG mice, comparable to WT mouse optic nerves (Fig. 3S, T). These results indicate that HCN channel-regulated midkine production is necessary for tumor progression, rather than initiation, but establish HCN channel activity as a targetable regulator of neuronal activity-dependent tumor progression.

### Arg1809Cys *Nf1*-conditional mutant mice do not develop plexiform neurofibromas

**Fig. 4:**
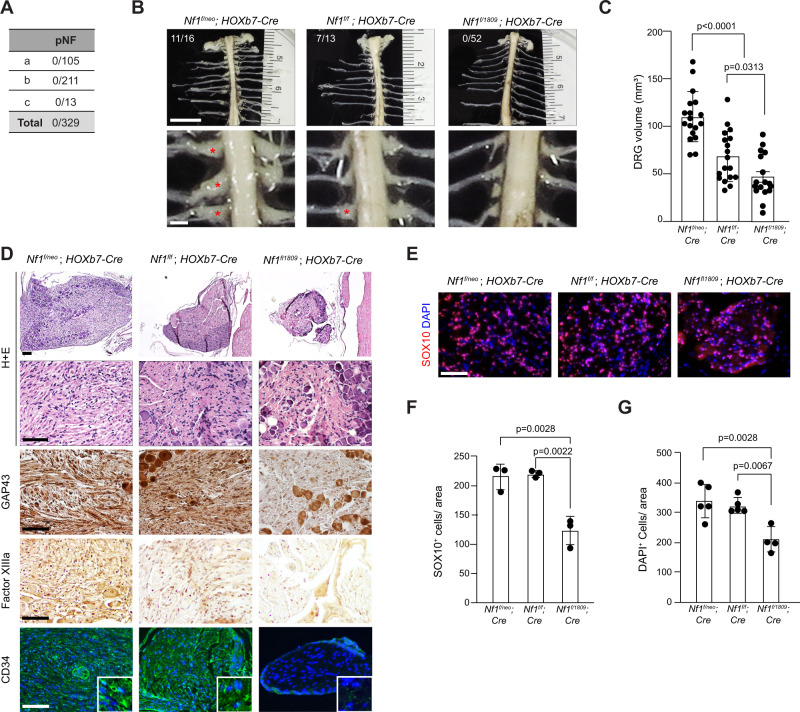
Arg1809Cys *Nf1-*mutant mice do not develop neurofibromas following somatic *Nf1* inactivation. **A** Incidence of peripheral nervous system tumors in NF1 patients harboring the c.5425 C > T *NF1* germline mutation. pNF: plexiform neurofibroma; (a)^44^, (b)^45^, (c)^40^. **B** Representative gross images (bright field) of spinal cords from 6-month-old *Nf1*^f/neo^*;* *Hoxb7-*Cre (*n* = 16), *Nf1*^f/f^; *Hoxb7*-Cre (*n* = 13), and *Nf1*^f/1809^ ; *Hoxb7-*Cre (*n* = 52) mice, showing (**C**) enlarged DRG (red asterisks) in *Nf1*^f/neo^; *Hoxb7-Cre* (*n* = 17; *P* < 0.0001) and *Nf1*^f/f^; *Hoxb7*-Cre mice (*n* = 17; *P* = 0.0313), but not in *Nf1*^f/1809^ ; *Hoxb7-*Cre mice (*n* = 17). Scale bars: 1 mm. The number of mice that formed pNFs is also shown in the top panels in (**B**). **D**, **E** Representative (**D**) H + E staining, GAP43, Factor XIIIa and CD34 staining, and (**E**) SOX10 and S100β, immunostaining. *n* = 4 for all groups. **F**, **G** Quantification of SOX10^+^ (*n* = 3 for all groups; *Nf1*^f/neo^; *Hoxb7-*Cre, *P* = 0.0028; *Nf1*^f/f^; *Hoxb7*-Cre, *P* = 0.0022) and DAPI^+^ cells (*Nf1*^f/neo^; *Hoxb7-*Cre, *n* = 5, *P* = 0.0028; *Nf1*^f/f^; *Hoxb7*-Cre, *n* = 5, *P* = 0.0067; *Nf1*^+/1809^; *Hoxb7-*Cre, *n* = 4) in DRGs. Scale bars, 50 µm. Data are presented as the mean ± SEM. One-way ANOVA with Tukey’s test for multiple comparison. Source data are provided as a Source Data file.

Since patients with the R1809C germline *NF1* gene mutation also do not develop plexiform neurofibromas (pNFs)^40,44,45^ (Fig. 4A), we engineered *Nf1*^+/1809^ mice with somatic loss of the conditional *Nf1* allele (*Nf1*^flox^) in Schwann cell progenitors, the cells of origin of pNFs^48,49^. The resulting *Nf1*^f/1809^; Hoxb7-Cre mice were analyzed and compared to conventional *Nf1*-mutant (*Nf1*^f/neo^; Hoxb7-Cre) mice that develop pNFs^50^. Unlike *Nf1*^f/neo^; Hoxb7-Cre mice (11/16), and *Nf1*^f/f^; Hoxb7-Cre mice (7/13), *Nf1*^f/1809^; Hoxb7-Cre mice did not develop pNFs at 6 months of age (0/52; Fig. 4B). Moreover, *Nf1*^f/1809^; Hoxb7-Cre mice exhibited neither enlarged DRGs (Fig. 4C) nor histological features of pNFs (Fig. 4D, E), and their DRGs contained fewer total cells, as well as fewer SOX10^+^ Schwann cell precursors, the cell of origin for these pNF tumors (Fig. 4F, G), demonstrating that mice harboring the *Nf1* R1809C mutation fail to develop pNFs.

### Tumor-associated *NF1-*mutant, but not *NF1*^+/R1089C^, sensory neurons produce COL1A2 in an activity-dependent manner

**Fig. 5:**
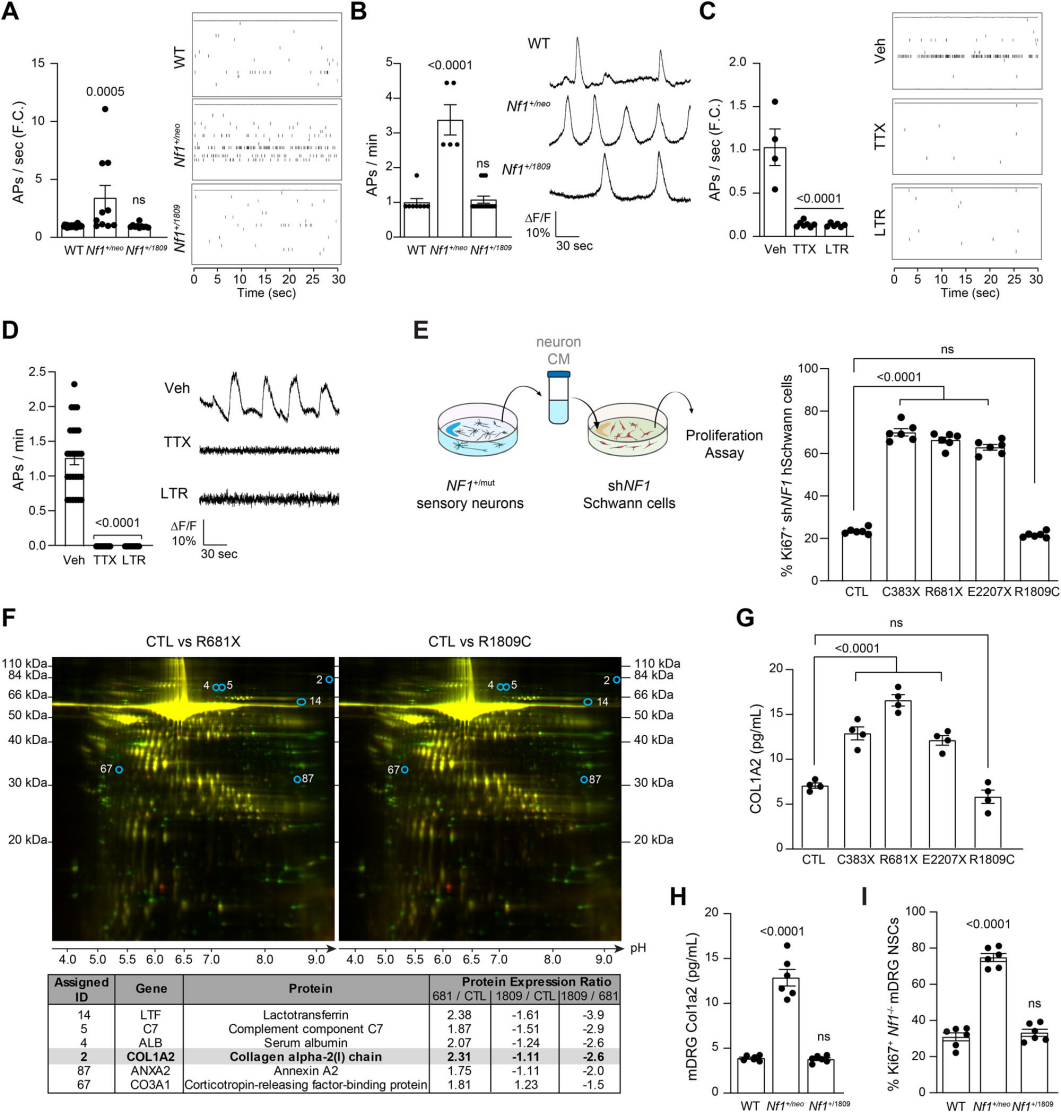
pNF-associated *NF1-*mutant PNS neurons exhibit increased activity and COL1A2-dependent preneoplastic *NF1*^*−/*^^−^ Schwann cell growth. **A**, **B** *Nf1*^+/neo^, but not *Nf1*^+/1809^, DRG neuron AP firing rates are elevated relative to WT DRG neurons, as measured by (**A**) multi-electrode array (WT, *n* = 24, *Nf1*^+/neo^, *n* = 10; *P* = 0.0005, *Nf1*^+/1809^ *n* = 10, ns), or (**B**) calcium imaging recordings (WT *n* = 8, *Nf1*^+/neo^ *n* = 5, *P* < 0.0001, *Nf1*^+/1809^ *n* = 14, ns). **C**, **D** TTX (1 µM) and lamotrigine (LTR; 200 µM) reduce *Nf1*^+/neo^ DRG neuron AP firing rate as measured by multi-electrode array (vehicle *n* = 4, TTX *n* = 7, *P* < 0.0001; LTR *n* = 6, *P* < 0.0001) and calcium imaging (vehicle *n* = 23, TTX *n* = 9, *P* < 0.0001, LTR *n* = 14, *P* < 0.0001). The right panels show representative (**A**, **C**) spike plots of entire multi-electrode array well recordings over 30 s, and (**B**, **D**) traces of neuronal activity over 3 min. **E** Schematic illustrating treatment of human sh*NF1* Schwann cells with hiPSC-sensory neuron conditioned media (CM). *NF1*-deficient Schwann cell proliferation is increased after treatment with *NF1*^C383X^, *NF1*^R681X^, and *NF1*^E2207X^ mutant neuron CM (*P* < 0.0001), but not *NF1*^R1809C^ neuron CM relative to controls (CTL). *n* = 6 for all groups. **F** Analytical comparison of 2D gel electrophoresis (top-to-bottom: decreasing molecular weight; left-to-right: decreasing acidity) of *NF1*^R681X^ (left) and *NF1*^R1809C^ (right) CM relative to CTL hiPSC-sensory neuron CM. Red dots indicate proteins with increased expression, green dots indicate proteins with decreased expression, and yellow dots indicate unaltered proteins in *NF1-*mutant sensory neuron CM relative to CTL neuron CM. The six proteins uniquely increased more than 1.5-fold in *NF1*^R681X^ hiPSC-sensory neuron CM relative to CTL, but not in *NF1*^R1809C^ CM, relative to CTL are circled in blue and are listed in the lower panel. Representative CM from CTL, *NF1*^R1809C^, and *NF1*^R681X^ sensory neurons was analyzed by 2D gel electrophoresis (*n* = 1). **G**, **H** COL1A2 levels are increased in (**G**) *NF1*^C383X^, *NF1*^R681X^, and *NF1*^E2207X^ mutant neuron CM (*P* < 0.0001), but not in *NF1*^R1809C^ neuron CM (*n* = 4 for all groups), as well as in (**H**) *Nf1*^+/neo^ mouse DRG neuron CM (*P* < 0.0001), but not in *Nf1*^+/1809^ mouse DRG neuron CM (*n* = 6 for all groups). **I** *Nf1*-deficient DRG-NSC proliferation is increased after treatment with *Nf1*^+/neo^ DRG neuron CM (*P* < 0.0001), but not *Nf1*^+/1809^ DRG neuron CM, relative to WT controls. *n* = 6 for all groups. Data are presented as the mean ± SEM. **A**–**E**, **G**–**I** One-way ANOVA with (**A**–**D**, **G**–**I**) Dunnett’s, or (**E**) Tukey’s multiple comparisons test. *P* values are indicated within each panel. ns, not significant. Source data are provided as a Source Data file.

We next sought to ascertain whether *Nf1*-mutant peripheral sensory neurons similarly exhibit increased activity. As such, we analyzed action potential firing rates of WT, *Nf1*^+/neo^, and *Nf1*^+/1809^ DRG neurons using multi-electrode array and calcium-imaging recordings (Fig. 5A, B). As observed in *Nf1*^+/neo^ CNS neurons, *Nf1*^+/neo^, but not *Nf1*^+/1809^, DRG neurons exhibited 3.4-fold increased action potential firing rates relative to WT controls (Fig. 5A, B and Supplementary Fig. 6A). Moreover, both TTX and lamotrigine reduced neuronal hyperexcitability ( > 85% reduction in action potential firing rate; Fig. 5C, D) relative to vehicle-treated controls. These results establish that *Nf1* mutation confers HCN channel activity-regulated sensory neuron hyperexcitability.

Based on our findings in the CNS, we hypothesized that PNS tumor (plexiform neurofibroma) growth is also dependent upon neuron activity-dependent paracrine factor secretion. Since neuronal trophic factors that mediate plexiform neurofibroma preneoplastic cell (*NF1*^*−/−*^ Schwann cells; sh*NF1* SCs, Supplementary Fig. 1A) growth have not yet been identified, we leveraged hiPSC-derived sensory neurons that harbor heterozygous *NF1* mutations found in patients with (c.1149 C  >  A, p.Cys381X; c.2041 C  >  T, pArg681X; c.6619 C  >  T, p.Gln2207X; Group 1) or without (c.5425 C  >  T; p.Arg1809Cys; Group 2) neurofibromas (Fig. 5E and Supplementary Fig. 5B, C). As Schwann cells are the proliferative neoplastic cells in neurofibromas, their in vitro proliferation was used as a proof-of-principle measure of their potential to proliferate within a neurofibroma in vivo. We found that conditioned media (CM) from group 1, but from not group 2, *NF1*-mutant neurons increased preneoplastic sh*NF1* Schwann cell proliferation (3.4–3.6-fold increase in Ki67^+^ Schwann cells; Fig. 5E and Supplementary Fig. 5D).

Leveraging these observations, we performed unbiased protein secretome analyses on CM from control, and representative sensory neurons from group 1 (*NF1*^R681X^) and group 2 (*NF1*^R1809C^; Fig. 5F and Supplementary Fig. 6B, C). The secreted proteins from both *NF1*-mutant neurons were compared to those of the controls and each differentially regulated protein was assigned an arbitrary identification number. From the 176 differentially regulated proteins, the expression of six proteins was uniquely increased more than 1.5-fold in the tumor-associated *NF1*^R681X^ CM but not in the non-tumor-associated *NF1*^R1809C^ CM relative to control CM (Fig. 5F). As a secondary validation, CM from independently generated sensory neurons was used to confirm the presence and concentration of the six identified proteins. Of these, only COL1A2 was elevated in the CM from the tumor-associated group 1, but not in the non-tumor-associated group 2, hiPSC-sensory neurons, as well as in mouse *Nf1*^+/neo^ but not *Nf1*^+/1809^ DRG neurons (2.4–3.2-fold increase; Fig. 5G, H and Supplementary Fig. 6D–H). Importantly, both *Nf1*^+/neo^ mouse DRG (Fig. 5I) and *NF1*^R681X^ hiPSC-sensory neuron CM (Supplementary Fig. 6I) increased *Nf1*^−*/*−^ DRG-NSCs (murine Schwann cell progenitors) proliferation (2-8–3.1-fold increase in %Ki67^+^ cells) relative to control and *Nf1*^+/1809^ or *NF1*^R1809C^ neuron CM. Notably, COL1A2 was uniquely expressed by *NF1*-mutant PNS, but not CNS, neurons (Supplementary Fig. 6J).

### COL1A2 is both necessary and sufficient for preneoplastic *NF1*-null Schwann cell proliferation in vitro

**Fig. 6:**
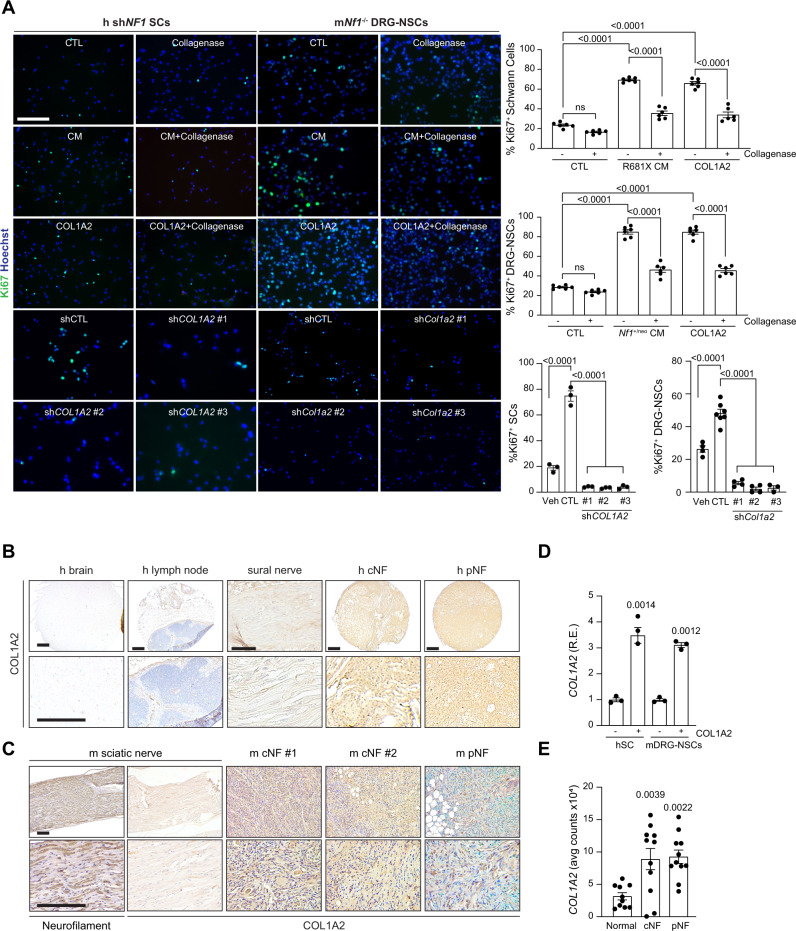
COL1A2 is necessary and sufficient for *NF1*-deficient Schwann cell growth in vitro. **A** Immunofluorescent staining and corresponding quantitation of Ki67^+^ human sh*NF1* Schwann cells (left) and *Nf1*^−*/−*^ mouse DRG–NSCs (right) following incubation with hiPSC-sensory neuron conditioned media (CM), with (h *P* = 0.0007; m *P* < 0.0001) and without (*P* < 0.0001) collagenase (*n* = 6 for all groups), COL1A2 alone with (h *P* = 0.0036; m *P* < 0.0001) and without (*P* < 0.0001) collagenase (*n* = 6 for all groups), as well as with and without control or short hairpins against *COL1A2* (*n* = 3 for all groups, *P* < 0.0001) or *Col1a2* (vehicle *n* = 4, control short hairpin *n* = 7, sh*Col1a2*-1 *n* = 4, sh *Col1a2*-2 *n* = 4, sh *Col1a2*-3 *n* = 3, *P* < 0.0001). **B**–**C** (**B**) Human and (**C**) mouse cutaneous (cNF) and plexiform neurofibromas (pNF) express COL1A2. Normal brain, lymph node and normal sural (human) or normal sciatic (mouse) nerves were negative for COL1A2 expression. Neurofilament was used as positive control for normal mouse nerve tissue. These data derive from a single-tissue microarray. **D** *COL1A2* RNA expression is increased in human sh*NF1* Schwann cells (left; *P* = 0.0014) and mouse *Nf1*^*−/−*^ DRG–NSCs (right; *P* = 0.0012) following COL1A2 treatment. *n* = 3 for all groups. **E** *COL1A2* RNA expression is increased in human Schwann cells isolated from human cNF (*P* = 0.0039) and pNF tumors (*P* = 0.0022) relative to controls. Normal *n* = 10, cNF *n* = 11, pNF *n* = 11. Data are presented as the mean ± SEM. **A**, **E** One-way ANOVA with (**A**) Tukey’s or (**E**) Dunnett’s multiple comparisons test, or (**D**) paired two-tailed Student *t* test. Scale bars, 50 µm. Source data are provided as a Source Data file.

To determine whether COL1A2 can increase *NF1*-deficient preneoplastic Schwann cell proliferation in vitro, human sh*NF1* SCs and murine *Nf1*^*−/−*^ DRG NSCs were treated with COL1A2 at the concentration quantified in sensory neuron CM (12.5 µg/mL). As such, COL1A2 treatment increased the proliferation of sh*NF1* SCs and *Nf1*^*−/−*^ DRG NSCs (2.5–2.9-fold increase in Ki67^+^ cells) to levels similar to *NF1*-mutant sensory neuron CM. The increase in proliferation conferred by *NF1*-mutant sensory neuron CM or COL1A2 alone was completely abrogated by neuron treatment with collagenase (Fig. 6A), as well as by genetic *COL1A2* short hairpin-mediated genetic reduction (sh*COL1A2* 1–3; 65.1% reduction, Supplementary Fig. 6K, L and Fig. 6A), or *Col1a2* (sh*Col1a2* 1–3*;* 70.2% reduction, Supplementary Fig. 6M, N and Fig. 6A). In addition, both human (Fig. 6B) and murine (Fig. 6C) cutaneous and plexiform neurofibromas exhibited strong COL1A2 immunoreactivity, in contrast to minimal expression in normal sural and sciatic nerves, lymph nodes, or brain. Similar to neuroligin-3 autocrine regulation of tumoral NLGN3 production^22–24^, incubation of sh*NF1* SCs and *Nf1*^*−/*−^ DRG NSCs with COL1A2 induced a feed-forward increase in *COL1A2* transcript levels (Fig. 6D). This feed-forward induction suggests a paracrine effect of neuronal COL1A2 on preneoplastic Schwann cell *COL1A2* transcription. Consistent with these findings, Schwann cells isolated from human cNFs or pNFs express higher levels of *COL1A2* relative to non-neoplastic SCs (Fig. 6E). Together, these findings establish COL1A2 as a unique neuronal-secreted factor critical for pNF-associated *NF1*^*−/−*^ neoplastic Schwann cell proliferation.

### COL1A2 secretion is neuronal activity-dependent

**Fig. 7:**
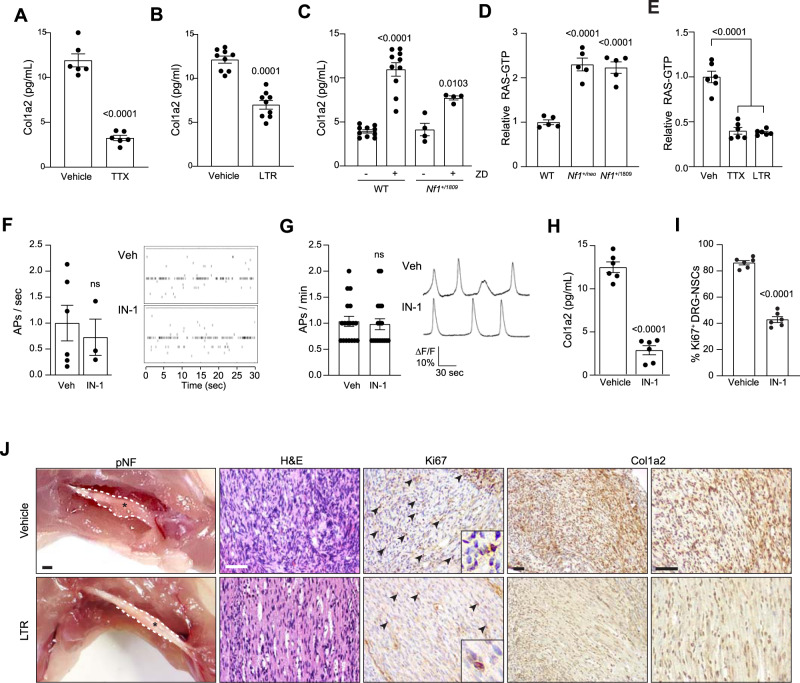
Col1a2 secretion is regulated by HCN channel-regulated sensory neuron activity. **A**, **B** TTX (1 µM; **A**; vehicle *n* = 6, TTX *n* = 6; *P* < 0.0001) and lamotrigine (LTR; 200 µM; **B**; vehicle *n* = 9, LTR *n* = 9; *P* = 0.0001) reduce *Nf1*^+/neo^ DRG neuron Col1a2 secretion by 73 and 47% relative to vehicle-treated controls. **C** ZD7288 (ZD; 30 µM) increases Col1a2 secretion in WT (*n* = 10 in both groups; *P* < 0.0001) and *Nf1*^+/1809^ (*n* = 4 in both groups; *P* = 0.0103) DRG neurons. **D** RAS activity is increased in both *Nf1*^+/neo^ and *Nf1*^+/1809^ DRG neurons relative to controls (*n* = 5 in all groups; *P* < 0.0001), (**E**) and is inhibited following TTX and LTR treatment (*n* = 6 in all groups; *P* < 0.0001). **F**, **G** IN-1 has no effect on DRG neuronal activity, as measured by (**F**) multi-electrode array (vehicle *n* = 6, IN-1 *n* = 3, ns not significant), or (**G**) calcium-imaging recordings (vehicle *n* = 18, IN-1 *n* = 18; ns, not significant). Right: representative (**F**) spike plots of entire multi-electrode array well recordings over 30 s, and (**G**) traces of neuronal activity over 3 min. **H** IN-1 reduces Col1a2 secretion by 77.9% in *Nf1*^+/neo^ DRG neurons. *n* = 6 for both groups, *P* = 0.0001. **I** IN-1 reduces proliferation by 50% in *Nf1*^*−/*−^ DRG–NSCs. *n* = 6 for both groups, *P* < 0.0001. **J** Lamotrigine treatment decreases pNF progression in vivo. Gross images and representative immunostaining of mouse pNFs demonstrate that LTR treatment reduces pNF size, partly restores neuronal histology (H&E), reduces proliferation (Ki67^+^ cells) and decreases Col1a2 production. Scale bars: gross anatomy images, 1 mm; sections, 100 µm. *n* = 5 for both groups. Data are represented as means ± SEM (**A**–**C**, **H**, **I**) using two-tailed paired Student’s *t* tests, (**F**, **G**) two-tailed unpaired *t* tests, or (**D**, **E**) one-way ANOVA with Dunnett’s post-test correction. *P* values are indicated within each panel. ns, not significant. Source data are provided as a Source Data file.

To determine whether neuronal excitability similarly controls PNS mitogen secretion, we analyzed Col1a2 in CM from TTX- and lamotrigine-treated *Nf1*^+/neo^ DRG neurons. Similar to midkine in their CNS counterparts, both TTX and lamotrigine reduced *Nf1*^+/neo^ DRG neuronal Col1a2 secretion (3.6-, 1.8-fold reduction, respectively; Fig. 7A, B), while ZD7288 increased DRG Col1a2 secretion in WT and *Nf1*^+/1809^ DRG neurons (2–2.8-fold increase; Fig. 7C). In addition, RAS activity was higher in both *Nf1*^+/neo^ and *Nf1*^+/1809^ DRG neurons (2.1-fold increase, Fig. 7D). The increased RAS activity in *Nf1*^+/neo^ DRG neurons was reduced following neuronal activity inhibition either by TTX or lamotrigine exposure (2.5–2.7-fold decrease; Fig. 7E). In addition, RAS inhibition had no effect on DRG neuronal activity (Fig. 7F, G), but reduced COL1A2 expression both in mouse (4.5-fold reduction, Fig. 7H) and human (2.6-fold reduction; Supplementary Fig. 6P) sensory neurons, and decreased *Nf1*^−*/*−^ DRG-NSC proliferation in vitro (Fig. 7I). These findings demonstrate that Col1a2 is secreted by tumor-associated *Nf1*^+/neo^ sensory neurons in an HCN channel activity-dependent manner.

Finally, to determine whether HCN channel function can govern pNF progression in vivo, mice harboring NF1-pNFs received intraperitoneal injections of lamotrigine for 6 weeks. HCN activation reduced pNF size, partly restored neuronal histology, and reduced both proliferation (Ki67^+^ cells), as well as Col1A2 immunoreactivity, within the tumors (Fig. 7J). Together, these data firmly establish that HCN channel-mediated sensory neuron Col1a2 production regulates pNF progression in vivo.

## Discussion

**Fig. 8:**
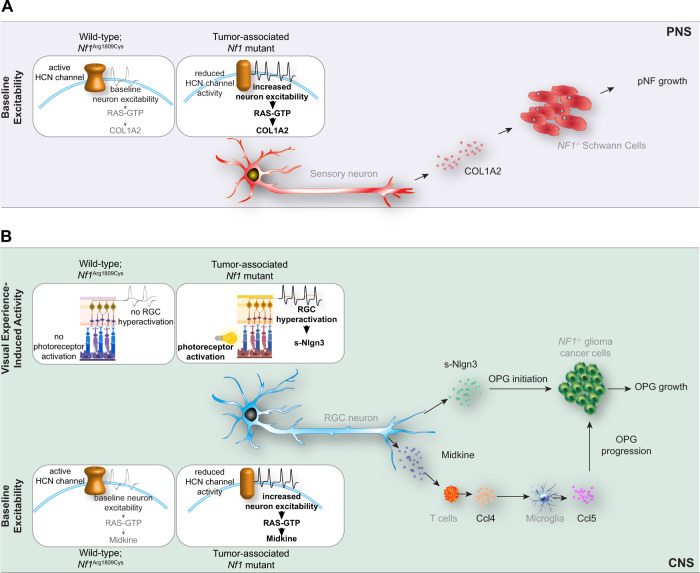
Proposed model for *NF1* mutation-induced, neuronal hyperexcitability-regulated low-grade tumor growth. **A** Tumor-associated *NF1*-mutant sensory neurons have increased baseline neuron excitability and deregulated HCN channel function, leading to elevated COL1A2 secretion. COL1A2, in turn, increases *NF1*^*−/*^^−^ Schwann cell proliferation to stimulate pNF growth. **B** Tumor-associated *NF1-*mutant retinal ganglion cell (RGC) activity is governed by two distinct mechanisms. First, visual experience (light)-induced activity enhances RGC production of soluble-Nlgn3 (s-Nlgn3), which drives OPG initiation and cell growth. Second, tumor-associated *NF1-*mutant RGCs have increased intrinsic baseline neuronal hyperexcitability, which is controlled by HCN channel function. Increased baseline HCN channel-regulated RGC excitability triggers increased midkine production to induce a T-cell (Ccl4) and microglial (Ccl5) signaling cascade that governs OPG progression and growth. PNS, peripheral nervous system, CNS, central nervous system, pNF, plexiform neurofibroma, OPG, optic pathway glioma. Small elements of this schematic were designed on BioRender.com. Source data are provided as a Source Data file.

Exploiting a unique, naturally occurring germline mutation in patients with the NF1 tumor predisposition syndrome who fail to develop neurofibromas or optic gliomas (Arg1809Cys), we employed hiPSCs and genetically engineered mice to identify two distinct mechanisms underlying neuronal activity regulation of nervous system tumor progression (Fig. 8).

In this study, and similar to what is observed in patients with NF1^40,44,45^, we first show that *Nf1*^+/1809^ mice do not form pNFs or OPGs. Consistent with the lack of tumor formation, Arg1809Cys-mutant neurons do not induce Adam10-mediated cleavage and shedding of Nlgn3, a growth factor required for murine *Nf1*-OPG initiation and growth^22^. In addition, we previously described a neuron-immune-cancer cell axis^7^, where neurons indirectly regulate *Nf1*-OPG progression through their effects on T-cell Ccl4-mediated induction of microglial growth factor (Ccl5) production. Since *Nf1*^+/1809^ T cells produce Ccl4 in response to midkine and *Nf1*^+/1809^ microglia produce Ccl5 in response to Ccl4, the Arg1809Cys mutation appears to operate at the level of the neuron, such that human and mouse neurons with this mutation fail to increase midkine expression or activate optic glioma-infiltrating T cells to drive *Nf1*-OPG progression. Importantly, tumor initiation may also be influenced by the germline *Nf1* mutation acting on the tumor cells of origin^51^, requiring investigations of the impact of the *NF1* Arg1809Cys mutation on third ventricle neural progenitors (OPG)^52^ and Sox10^+^, GAP43^+^ Schwann cell precursors (neurofibroma)^48–50,53^.

Second, we identified COL1A2 as a sensory neuron-derived paracrine factor important for *NF1*-deficient Schwann cell proliferation. Of note, Schwann cells are the neoplastic cells of two distinct types of tumors, neurofibromas, and schwannomas, which differ both in pathology and immunohistochemical profiles. Specifically, neurofibromas, which occur both sporadically and in the setting of NF1, are heterogeneous tumors with small and wavy nuclei, excess “shredded” type collagen, and are immunopositive for neurofilament expression. In contrast, schwannomas arising either sporadically or in patients with neurofibromatosis type 2 (NF2) and Schwannomatosis are encapsulated tumors with more homogeneous Schwann cell proliferation, larger nuclear sizes, and the presence of hyalinized vessels^54^. The importance of collagen to neurofibroma-associated Schwann cell growth is underscored by the observation that collagen accounts for the majority of the extracellular matrix in human neurofibromas and as much as 50% of neurofibroma dry weight^55^. While type 1 collagens increase Schwann cell and Schwann cell progenitor adhesion, survival, and proliferation^56–58^, we show that *NF1* mutation in human and mouse peripheral sensory neurons induces activity-dependent production of COL1A2, which, similar to NLGN3 in the brain^22–24^, induces a feed-forward loop of *COL1A2* transcription in Schwann cells and Schwann cell progenitors, resulting in elevated tumoral collagen levels. While the abundance of collagen and its production by other cell types (fibroblasts) in neurofibromas^11^ prompted human clinical trials with broad-spectrum anti-fibrotic agents, like Pirfenidone, no efficacy was observed^59^, possibly due to the low abundance of collagen-synthesizing fibroblasts in pNFs^60^. Ongoing studies are focused on determining whether targeting of sensory neuron-specific COL1A2 production will reduce neurofibroma growth.

Third, examination of *NF1*^+/1809^ neurons revealed unique non-RAS functions for the *NF1* protein, neurofibromin. In this regard, *NF1*^+/1809^ neurons exhibit elevated RAS activity, similar to neurons with *NF1* mutations from patients who develop neurofibromas or optic gliomas. However, *Nf1*^+/1809^ neurons do not exhibit increased action potential firing rates necessary to drive Nlgn3 and midkine (retinal ganglion cells) or COL1A2 (sensory neurons) secretion. These findings uncouple RAS regulation from the control of baseline neuronal excitability, and suggest that other non-RAS-dependent mechanisms account for these neurofibromin-regulated effects in neurons. While there are a few examples of non-RAS-dependent functions for neurofibromin^37,47,61–63^ additional studies will be necessary to determine whether the *NF1* Arg1809Cys mutation, located within the PH-like domain of neurofibromin^64^, affects the conformation of the protein relative to neurofibromin dimerization^65^, secondary structure^40,64^, or associations with other neurofibromin-binding partners in neurons^66–68^.

Fourth, we demonstrate that *NF1* mutation regulates neuronal hyperexcitability intrinsically through HCN channel function, and this hyperexcitability is evident in visual experience-evoked activation. The finding of hyperexcitability parallels prior studies of *Nf1*^+/neo^ sensory neurons, which have greater numbers of action potentials, lower firing thresholds, lower rheobase currents, and shorter firing latencies^69^. Herein, we demonstrate that baseline *NF1* regulation of neuronal hyperexcitability involves dysregulated HCN channel function (midkine, COL1A2 production)^37^. HCN channels are voltage-operated cation channels expressed in RGC^70,71^. and DRG neurons^71–73^. Inhibition of HCN channel signaling with antagonists, such as ZD7288, increases neuron firing rates in vivo^74^, paralleling the effects of HCN channel agonist (LTR) and antagonist (ZD7288) treatments on CNS and PNS neuron hyperexcitability and activity-dependent regulation of midkine and Col1a2 expression. Additionally, RGC hyperexcitability in the context of visual experience and consequent Adam10/Nlgn3 production are required for *Nf1*-OPG initiation, such that *Nf1*-optic glioma-prone mice do not develop tumors if reared in the dark during critical periods of tumorigenesis, or if *Nlgn3* is genetically or pharmacologically blocked^22^. As light-induced activity did not affect RGC midkine expression and Adam10/Nlgn3 production was not dependent on HCN channel function, we postulate that *Nf1*-OPG initiation relies on light-mediated RGC activation and Nlgn3 shedding, whereas OPG progression requires both Nlgn3 shedding and HCN channel-regulated baseline neuronal activity and midkine production.

Taken together, the findings reported herein advance our growing appreciation of neurons as active participants in tumor biology. While we conclusively establish that neuronal hyperexcitability drives mouse *Nf1* OPG and pNF progression, future work using genetically engineered mouse strains and ectopic gene delivery methods will be necessary to demonstrate that midkine and Col1a2 expression are solely sufficient to maintain murine OPG and pNF growth in vivo, respectively. Additional efforts will include the identification of key modulators of central and peripheral nervous system neuron-dependent tumorigenesis. This presents unique opportunities to repurpose FDA-approved compounds that target neuron-produced mitogens (e.g., collagenase^75^) or HCN channels (e.g., Lamotrigine; Ivabradine^76,77^) for the treatment of NF1-associated nervous system tumors, expanding the toolbox for targeting neuron-low-grade tumor interactions in cancer.

## Methods

All experiments were performed in compliance with active Animal Studies Committee protocols at Washington University and UT Southwestern.

### Mice

All experiments were performed under active Animal Studies Committee protocols at Washington University School of Medicine (Washington University in St Louis Institutional Animal Care and Use Committee) and UT Southwestern (UT Southwestern Institutional Animal Care and Use Committee). According to these ethics committees, any animals with compromised motion/eating habits or an unhealthy appearance are euthanized. No animals were euthanized due to their tumor burden or as a result of the treatments performed in this study. Mice were maintained on a 12 light/ dark cycle in a barrier facility, at 21 °C and 55% humidity, and had ad libitum access to food and water. Heterozygous *Nf1* c.5425 C  >  T*;* Arg1809Cys-mutant mice were generated by CRISPR/ Cas9 engineering directly into C57Bl/6J embryos, resulting in mice with one wild-type *Nf1* allele and one missense R1809C mutation. The mutation was confirmed by direct sequencing (IDT Technologies). R1809C *Nf1*-mutant mice, as well as heterozygous R681X^35,36^ and c.3827G > C^43^ *Nf1*-mutant mice were backcrossed to C57Bl/6J and wild-type littermates were used as controls. For pNF studies, mice were generated with the R1809C mutation or a neomycin cassette inserted in exon 31^46^ as the germline *Nf1* allele and somatic *Nf1* inactivation in Hoxb7-Cre cells^78^ (*Nf1*^flox/-^;Hoxb7-Cre; *Nf1*^flox/1809^*;Hoxb7-*Cre). In addition, conditional knockout *Nf1*^flox/flox^; *Hoxb7-*Cre mice were used. Optic glioma-prone mice were generated with the R1809C mutation or a neomycin cassette inserted in exon 31^46^ as the germline *Nf1* allele and somatic *Nf1* inactivation in neuroglial progenitor cells^79^ (*Nf1*^f/1809^; hGFAP-Cre or *Nf1*^f/neo^; hGFAP-Cre mice). Littermate *Nf1*^flox/flox^ mice were used as controls. For light/dark-rearing experiments, eight *Nf1*^+/neo^ mice were reared in the dark for 4 weeks from 4 weeks of age. Eight littermate controls were reared in normal 12 h light/dark cycles. For in vivo lamotrigine treatment of NF1-pNFs, 8-week-old athymic nude mice (Charles River, Stock No. 490) underwent surgery to implant pNF progenitor cells. Mice of both sexes were randomly assigned to all experimental groups without bias, and the investigators were blinded until the final data analysis during all of the experiments.

### Human-induced pluripotent stem cells and neuronal differentiation

NF1 patient heterozygous germline *NF1* gene (Transcript ID NM_000267) mutations were CRISPR/Cas9-engineered into a single commercially available male control human iPSC line (BJFF.6) by the Washington University Genome Engineering and iPSC Core Facility (GEiC). hiPSCs were authenticated based on morphology, as well as by immunocytochemical expression of pluripotency markers. Human iPSCs were differentiated into neural progenitor cells after 7 days of embryoid body formation (StemDiff Neural induction media; STEMCELL Technologies), followed by embryoid body dissociation and plating in PLO/Laminin-coated flasks in 50% DMEM/F12, 50% Neurobasal medium supplemented with N2, B27, 2 mM GlutaMAX (all Gibco), 10 ng/mL hLIF, 3 μM CHIR99021 and 2 μM SB431541 (all STEMCELL Technologies). NPCs were subsequently differentiated either into excitatory CNS neurons following incubation in neurobasal medium supplemented with B27, 2 mM glutamine, and 50 U/mL penicillin/streptomycin for a minimum of 2 weeks, or into GABAergic CNS neurons following incubation in neurobasal medium supplemented with 1 μM cAMP, 10 ng/mL BDNF, 10 ng /mL GDNF, and 10 ng/mL IGF1^47^. For sensory neuron differentiation, iPSCs were incubated for 8 days in DMEM/F12 supplemented with LDN-193189, CHIR99021, A83-01, RO4929097, SU5402, retinoic acid, and 10% knockout serum replacement followed by 4 weeks of neurobasal medium supplemented with NT3, nerve growth factor, brain-derived neurotrophic factor, and glial-derived neurotrophic factor. No commonly misidentified cell lines were used in this study.

### Spinal cord dissection and optic nerve processing

Mice were transcardially perfused at 3 months of age with Ringer’s solution and 4% paraformaldehyde. Whole spinal cords were isolated following the removal of gross and muscle tissue and the breaking of vertebral column bones under a microdissection microscope. The entire spinal cord and peripheral nerves were rinsed and fixed in 10% formalin-buffered solution. DRG diameters were measured as previously reported^53,80^ and tumor volumes were calculated as volume = length × width^2^ × 0.52, which approximates the volume of a spheroid^53,80^. Optic nerves were isolated, imaged using a Leica DFC 3000 G camera, and their volumes were calculated as previously described^81^. Using ImageJ, four diameter measurements were taken to estimate the thickness of each optic nerve beginning at the chiasm (D_0_), at 150 (D_150_), 300 (D_300_), and 450 µm (D_450_) anterior to the chiasm. The following equation was used to calculate the estimated optic nerve volume in each of the three sections, the sum of which was ultimately used to calculate the total optic nerve volume: V_1_ = 1/12 *π*h (D_0_^2^ + D_0_D_150_ + D_150_^2^).

### Primary hippocampal, RGC, and DRG neuron cell culture

Primary neuron cultures were generated from postnatal day 4–10 WT, *Nf1*^+/neo^ or *Nf1*^+/1809^ mice. Hippocampi were dissected in Hibernate-A (Gibco) and primary hippocampal neurons were established after papain dissociation, following the manufacturer’s instructions (Worthington). Hippocampal neurons were grown for 7 days prior to analyses. Retinae were dissected in Hibernate-A (Gibco), dissociated in papain (Worthington) and ovomucoid inhibitor (Worthington) before being filtered with CD11b magnetic beads (Miltenyi Biotech) to deplete microglia. The remaining RGCs were plated on poly-D-lysine (Sigma)-coated plates and incubated in neurobasal media supplemented with N2, T3, transferrin, BSA, progesterone, putrescine, sodium selenite, l-glutamine, insulin, N-acetyl cysteine, and forskolin. RGC neurons were grown for 4 days prior to analyses. DRG tissues were isolated in HBSS (Gibco), dissociated in papain (Worthington biochemical) and collagenase type I (STEMCELL Technologies), prior to being strained (70 µm), plated in fibronectin (Fisher)-coated plates, and incubated in 10% fetal bovine serum in DMEM (Gibco). DRG neurons were grown for 7 days.

### T cell and microglia isolation

Four to six-week-old WT and *Nf1*^+/1809^ mouse spleens were homogenized into single-cell suspensions by digestion in PBS containing 0.1% BSA and 0.6% sodium citrate. The homogenates were subsequently washed and incubated with 120 Kunitz units of DNase I for 15 min following red blood cell lysis (eBioscience). Cells were then filtered through a 30 µM cell strainer to obtain a single-cell suspension. T cells were maintained at 2.5 × 10^6^ cells ml^−1^ in RPMI-1640 medium supplemented with 10% FBS and 1% penicillin/streptomycin. T cells were treated with 100 ng/µL midkine (R&D Systems) for 48 h. Microglia isolation was performed on 4–6-week-old WT and *Nf1*^+/1809^ mouse brains using the multi-tissue dissociation kit (Miltenyi Biochemicals) following published protocols^7^. The resulting cells, microglia attached to a monolayer of astrocytes, were maintained in minimal essential medium supplemented with 1 mM l-glutamine, 1 mM sodium pyruvate, 0.6% D-( + )-glucose, 1 ng/ml GM-CSF, 100 μg/ml P/S, and 10% FBS. From 11 days in vitro onwards, the cells were incubated in medium without GM-CSF and at 13 days in vitro, the cells were treated with 6000 pg/mL of recombinant Ccl4 (R&D Systems) for 24 h. At 14 days in vitro, the microglia were mechanically dissociated from the astrocyte layer by gentle shaking (200 g, 5 h, 37 °C). T-cell and microglia conditioned media were collected for subsequent ELISA experiments, both from control and treated cells following 22 µM filtration.

### sh*NF1* Schwann cell and *Nf1*^−/−^ DRG-NSC cultures

Normal human Schwann cells (Sciencell) were incubated in SCM (Sciencell) on PDL-coated plates following the manufacturer’s instructions and were infected with sh*NF1* 1–3 lentiviral particles (Sigma; 39714, 39715, 39717). *NF1* knockdown was confirmed by western blotting. *Nf1*^flox/flox^; *Cre* (*Nf1*^*−/−*^*)* DRG dorsal nerve root sphere cells (DRG-NSCs) were isolated from E13.5 embryo DRG/nerve roots^48^, and were infected with Ad-CMV-Cre. DRG-NSCs were incubated in DMEM supplemented with heparin, glucose, HEPES, L-glutamine, N2, B27, sodium carbonate, EGF and bFGF in ultralow cell attachment flasks, or fibronectin-coated flasks for 2D cell proliferation assays.

### Sensory neuron conditioned media protein analysis and validation of candidate proteins

Control, *NF1*^*+/R*1809C^ or *NF1*^+/R681X^ sensory neurons were washed with PBS and were incubated with artificial cerebral spinal fluid (aCSF) for 24 h prior to collecting conditioned media (CM). The media was treated with protease inhibitors (Cell Signaling Technologies), was snap frozen, and sent to Applied Biomics for 2D gel electrophoresis analysis. The conditioned media was run on a 2D electrophoresis gel and the proteins were separated by size and pH, as per the vendor’s specifications. The resulting digital images of the 2D gels of CTL, *NF1*^+/R681X^ and *NF1*^+/R1809C^ conditioned media (Supplementary Fig. 6B) were digitally superimposed pairwise by Applied Biomics (CTL vs *NF1*^+/R681X^ and CTL vs *NF1*^+/R1809C^) in order to detect differentially expressed proteins between each of the *NF1*-mutant neurons and the controls. In total, 176 dots (proteins) were upregulated or downregulated more than 1.5-fold relative to the CTL conditioned media, each dot was assigned a random identification number, and the intensity of the relative expression of each protein was translated into numerical values by the vendor. From the 176 differentially regulated proteins, only six (circled in blue; Fig. 5E) were upregulated more than 1.5-fold in *NF1*^+/R681X^ but not *NF1*^+/R1809C^ relative to CTL sensory neuron CM. As such, the identity of these six proteins alone was determined by mass spectrometry by Applied Biomics, following vendor specifications (Source data). No large-scale mass spectrometry or raw proteomics data was generated for these analyses. The concentration of each of these six identified proteins was assayed in independently generated CTL and *NF1*-mutant Schwann cell growth-promoting (*NF1*^C383X^, *NF1*^R681X^, *NF1*^E2207X^) and NF1-mutant non-Schwann cell growth-promoting (*NF1*^R1809C^) sensory neurons (Fig. 5F and Supplementary Fig. 6D–H) conditioned media by respective ELISA assays.

### Small-molecule treatments

A subset of mouse and human CNS and PNS neurons were treated with tetrodotoxin (TTX; 1 µM), pan-RAS inhibitor IN-1 (1 µM), lamotrigine (LTR; 200 µM), or ZD7288 (ZD; 30 µM) for 3 min prior to collection of cells or conditioned media. A subset of sh*NF1* SCs and *Nf1*^−/−^ DRG NSCs were treated with collagenase (0.001 U/mL), human COL1A2 (12.5 pg/mL), or mouse Col1a2 (12.5 pg/mL) for 24 h.

### Multi-electrode array (MEA) recordings and analyses

Primary hippocampal (300,000 cells/well), RGC (300,000 cells/well), or DRG neurons (150,000 cells/well) from each of the strains assayed (WT*, Nf1*^+/neo^, *Nf1*^+/1809^) were plated on AXION Biosystems 48-well MEA plates and grown for 10 days in their respective optimal growth media. Each well included neurons from a single mouse. A minimum of six individual mice originating from a minimum of three independent litters were analyzed. Neurons isolated from each animal were plated in a minimum of triplicate technical replicate wells of the MEA plate. For experiments involving pharmacological treatments, neurons isolated from each *Nf1*^+/neo^ mouse were plated in a minimum of six individual wells, with a minimum of three wells serving as the vehicle-treated controls and a minimum of three wells as the treated cohort. All efforts were taken to ensure even spreading of the neurons throughout each well.

Not all 16 electrodes present within each well were within the optimal proximity to neurons and as such not all electrodes detected action potentials (APs). To account for this variation, all metrics were normalized to the number of the active electrodes only. In addition, as the number of active electrodes/well varied between technical replicates of each animal, the AP firing rate of all the replicate wells of each animal was averaged. As such, each data point graphed represents the average of all technical replicates for each given animal. All neurons were recorded for 3 min at a 4.5 standard deviation threshold level and 5000 Hz as a digital filter using AXION Biosystems integrated studio (AxIS) version 2.5.1 software. AP firing rates were calculated from the total number of APs/3 min and are represented as APs/min, only accounting for active electrodes. Representative traces of action potentials were extracted using the AXION Biosystems neural metric tool and Offline sorter x64 version 4 software.

### Calcium imaging of neurons

Primary RGC (150,000 neurons/well) or DRG (75,000 neurons/well) neurons were plated onto poly-D-lysine and laminin-coated 96-well plates for 10 days. At 10 days, the cells were treated with Fluo-8/AM (1345980-40-6, AAT Bioquest), PowerLoad (P10020, ThermoFisher) and Probenecid (P36400; ThermoFisher) for 30 min at 37 °C and for another 30 min at room temperature. The neurons were subsequently washed with HBSS and incubated for a minimum of 10 min in fresh culture medium supplemented with 5% neuro-background suppressor (F10489; ThermoFisher). The neurons were imaged on a Nikon spinning disk upright epi-fluorescence confocal microscope equipped with a ×10 dry objective, and a 488 nm wavelength laser was used for wide-field imaging. The neurons were stimulated by a Ti LAPP DMD (Deformable Mirror Device) LED source for ultrafast photo-stimulation, with 0.1 mW applied during each recording for Fluo-8 excitation. Fluo-8 images were collected at 15 Hz (2048 × 2048 pixels, 1 × 1 mm) and the duration of each region of interest (ROI) was limited to 10 min. The fluorescence intensity and optical response to depolarizing membrane potential transients (ΔF/F) were calculated in Matlab programming environment to generate single-neuron activity traces. The ΔF/F threshold was set at 4 standard deviation beyond baseline fluorescence. Following data acquisition, the duration and shape of each AP spike were compared by merging all the spikes in the same time window. Neurons from each animal were seeded in six wells and a minimum of three neurons were recorded per well. Data recorded from a minimum of 18 neurons per animal were averaged. Each data point represents a single animal.

### Immunohistochemistry and Immunocytochemistry

All spinal cord and optic nerve fixed tissues as well as human brain tissue, lymph nodes, normal sural nerve, cutaneous neurofibromas or plexiform neurofibromas, and mouse sciatic nerve, cutaneous or plexiform neurofibromas were paraffin-embedded, serially sectioned (5 μm) and immunostained with GFAP, Iba1, Ki67, CD3, Midkine, GAP43, CD34, Factor XIIIa, SOX10, neurofilament-200, and Col1a2 (Supplementary Table 1). Immunohistochemical staining was performed using the Vectastain ABC kit (Vector Laboratories) and appropriate biotinylated secondary antibodies (Vector Laboratories). Hematoxylin and eosin (H&E) staining was performed following the manufacturer’s instructions (StatLab). Primary RGCs, hippocampal neurons, DRG neurons, sh*NF1* Schwann cells, and hiPSC-sensory neurons were immunostained with appropriate primary (RGCs: Rbpms, Tuj-1; hippocampal neurons: GAD65, Glutamine synthetase, Tuj-1; DRG neurons: Peripherin, ISL1, Tuj-1; SCs: EGR2, S100β, OCT6, SOX10; sensory neurons: peripherin, BRN3A, SMI32, ISL1, p75NTR, Nestin, Tuj-1) and secondary Alexa-fluor-conjugated antibodies (Supplementary Table 1). Images were acquired using Image Studio Lite Version 5.2 software, and LAS AF Lite 3.2.0 software and analyzed using ImageJ 1.53a software, as well as Adobe Photoshop version 21.1.1.

### RAS, midkine, COL1A2, Ccl4, Ccl5 ELISA assays

RAS activity (ThermoFisher), COL1A2 (Fisher Scientific,), Ccl4 (R&D Systems), Ccl5 (Fisher), and Midkine (mouse; LSBio; human; Abcam) ELISAs were performed on homogenized cell pellets (RAS-GTP) or filtered (0.22 µm) conditioned media (COL1A2, Ccl4, Ccl5, Midkine) following the manufacturer’s instructions. Each assay was performed using a minimum of four independently generated biological replicates. Data from all of these colorimetric assays were collected on a Bio-Rad iMark microplate reader and analyzed using MPM6 v6.3 (Bio-Rad Laboratories) software.

### Western blotting

Western blotting was performed on snap-frozen cells and tissues. Samples were lysed in RIPA buffer (Fisher) supplemented with a protease inhibitor cocktail (Cell Signaling) and were blotted using appropriate primary (s-Nlgn3, neurofilament-200, peripherin, BRN3A, ISL1, CALCA, α-tubulin, β-actin; Supplementary Table 1) and NIR-conjugated secondary antibodies (Licor). Images were captured and analyzed using the Li-Cor Image Studio Lite Version 5.2 software and are representative of more than three independently generated biological replicates.

### Quantitative real-time PCR

Total RNA was extracted following the manufacturer’s instructions (QIAGEN) and reverse-transcribed using a high-capacity cDNA reverse transcription kit (Applied Biosystems) qPCR was performed using TaqMan gene expression assays (*Mdk*, *Col1a2*, *COL1A2*, *Nlgn3, Adam10, NLGN3, ADAM10*, *Hcn1-4*; Supplementary Table 2) and TaqMan Fast Advanced Master Mix (Applied Biosystems) according to the manufacturer’s instructions. All reactions were performed using the Bio-Rad CFX96 Real-Time PCR system equipped with Bio-Rad CFX Manager 3.1 software. Gene expression levels of technical replicates were estimated by ΔΔCt method using *GAPDH* or *Gapdh* (Supplementary Table 2) as reference genes.

### In vivo mouse lovastatin and lamotrigine treatments

In total, 17 *Nf1*^flox/neo^; *GFAP*-Cre (*Nf1*-OPG) mice were intraperitoneally administered vehicle (saline in 1% methylcellulose; *n* = 9) or 25 mg/ Kg body weight lamotrigine (Selleckchem; *n* = 8) from four to six weeks of age, three times a week. The mice were then aged to 12 weeks for optic nerve and RGC analysis. For lovastatin treatments, 20 *Nf1*-OPG mice were administered with 10 mg/kg/day lovastatin (Santa Cruz Biotechnologies; *n* = 10) or vehicle (saline in 1% methylcellulose; *n* = 10) by oral gavage for 4 weeks, 5 days a week, beginning at 12 weeks of age, for 8 weeks. The mice were analyzed at 20 weeks of age.

For pNF analyses, *Nf1*^*−/−*^ DRG-NSCs were implanted in sciatic nerves of 8-week-old athymic nude mice as previously described^49^. Briefly, the mice underwent surgery to create a pocket by displacing the quadriceps muscle and exposing their sciatic nerve. In all, 1 × 10^6^ *Nf1*^*−/*^^−^ DRG-NSCs were implanted in the pocket around the sciatic nerve, such that the cells could be in direct contact with the nerve before the muscle and skin were sutured. Following recovery from the surgery, the mice were intraperitoneally administered vehicle (saline in 1% methylcellulose; *n* = 5) or 25 mg/kg body weight lamotrigine (Selleckchem; *n* = 5) three times a week for 6 weeks prior to histological analysis.

### Published RNA database analysis

The analysis for this paper was generated using Partek Flow software, version 10.0 using publicly available datasets (GEO: GSE14038; https://www.ncbi.nlm.nih.gov/geo/query/acc.cgi?acc=GSE14038, Supplementary Table 3). RNA-seq reads were aligned to the Ensembl release 100 top-level assembly with STAR version 2.7.8a. Gene counts and isoform expression were derived from Ensembl output. Sequencing performance was assessed for the total number of aligned reads, total number of uniquely aligned reads, and features detected. Normalization size factors were calculated for all gene counts by CPM to adjust for differences in sequencing depth. Genes not expressed on average with greater than two count-per-million were excluded from further analysis. Gene-specific analysis was then performed using the lognormal with shrinkage model (limma-trend method) to analyze for expression differences between conditions.

### Short hairpin constructs, lentiviral production, and neuronal infection

Human sh*COL1A2* and mouse sh*Col1a2* lentiviral particles (TRCN0000090043; TRCN0000090045; TRCN0000335210) were generated as previously described^61^. *NF1*^+/R681X^ or *Nf1*^+/neo^ sensory neurons were infected with three independent sh*COL1A2* lentiviral particles or shRNA scrambled control particles (sc-108080; Santa Cruz Biotechnology) for 24 h. Neuronal media was refreshed and conditioned media was collected for subsequent assays 48–72 h post infection.

### Quantification and statistical analysis

All statistical tests were performed using GraphPad Prism software (versions v5, v_8.2.1, and v_9.3.1). Paired or unpaired two-tailed Student’s *t* tests or one-way analysis of variance (ANOVA) with Tukey’s, Dunnett’s, or Bonferroni post-test correction using GraphPad Prism 5 software. Statistical significance was set at *P* < 0.05, and individual p values are indicated within each graphical figure. A minimum of three independently generated biological replicates was employed for each of the analyses. Numbers (*n*) are noted for each individual analysis.

### Reporting summary

Further information on research design is available in the Nature Research Reporting Summary linked to this article.

## Supplementary information

Supplementary Information

Description of Additional Supplementary Files

Reporting Summary

Supplementary Movie 1.

Supplementary Movie 2.

Supplementary Movie 3.

Supplementary Movie 4.

Supplementary Movie 5.

Supplementary Movie 6.

Supplementary Movie 7.

Supplementary Movie 8.

Supplementary Movie 9.

Supplementary Movie 10.

Supplementary Movie 11.

Supplementary Movie 12.

Supplementary Movies 13.

Supplementary Movie 14.

Supplementary Movie 15.

Supplementary Movie 16.

Supplementary Movie 17.

## Data Availability

Publicly available RNA sequencing datasets (GEO: GSE14038) were analyzed in this study. Source data are provided with this paper.

## Source data

Source Data

## References

[1] Joyce JA (2005). Therapeutic targeting of the tumor microenvironment. Cancer Cell.

[2] Ungefroren H, Sebens S, Seidl D, Lehnert H, Hass R (2011). Interaction of tumor cells with the microenvironment. Cell Commun. Signal.

[3] Hu M (2005). Distinct epigenetic changes in the stromal cells of breast cancers. Nat. Genet..

[4] Gorzalczany Y, Akiva E, Klein O, Merimsky O, Sagi-Eisenberg R (2017). Mast cells are directly activated by contact with cancer cells by a mechanism involving autocrine formation of adenosine and autocrine/paracrine signaling of the adenosine A3 receptor. Cancer Lett..

[5] Ferner RE, Gutmann DH (2013). Neurofibromatosis type 1 (NF1): diagnosis and management. Handb. Clin. Neurol..

[6] Daginakatte GC, Gutmann DH (2007). Neurofibromatosis-1 (Nf1) heterozygous brain microglia elaborate paracrine factors that promote Nf1-deficient astrocyte and glioma growth. Hum. Mol. Genet..

[7] Guo X (2020). Midkine activation of CD8(+) T cells establishes a neuron-immune-cancer axis responsible for low-grade glioma growth. Nat. Commun..

[8] Pan Y, Smithson LJ, Ma Y, Hambardzumyan D, Gutmann DH (2017). Ccl5 establishes an autocrine high-grade glioma growth regulatory circuit critical for mesenchymal glioblastoma survival. Oncotarget.

[9] Mashour GA (2001). The angiogenic factor midkine is aberrantly expressed in NF1-deficient Schwann cells and is a mitogen for neurofibroma-derived cells. Oncogene.

[10] Yang FC (2008). Nf1-dependent tumors require a microenvironment containing Nf1+/− and c-kit-dependent bone marrow. Cell.

[11] Yang FC (2006). Nf1+/− mast cells induce neurofibroma like phenotypes through secreted TGF-beta signaling. Hum. Mol. Genet.

[12] Chen S (2010). Nf1-/- Schwann cell-conditioned medium modulates mast cell degranulation by c-Kit-mediated hyperactivation of phosphatidylinositol 3-kinase. Am. J. Pathol..

[13] Fletcher JS, Pundavela J, Ratner N (2020). After Nf1 loss in Schwann cells, inflammation drives neurofibroma formation. Neurooncol Adv..

[14] Fletcher, J. S. et al. Cxcr3-expressing leukocytes are necessary for neurofibroma formation in mice. *JCI Insight* **4**, 10.1172/jci.insight.98601 (2019). 10.1172/jci.insight.98601 PMC6413799 30728335

[15] Fletcher JS (2019). STAT3 inhibition reduces macrophage number and tumor growth in neurofibroma. Oncogene.

[16] Prada CE (2013). Neurofibroma-associated macrophages play roles in tumor growth and response to pharmacological inhibition. Acta Neuropathol..

[17] Guo X, Pan Y, Gutmann DH (2019). Genetic and genomic alterations differentially dictate low-grade glioma growth through cancer stem cell-specific chemokine recruitment of T cells and microglia. Neuro Oncol..

[18] Pan Y (2018). Athymic mice reveal a requirement for T-cell-microglia interactions in establishing a microenvironment supportive of Nf1 low-grade glioma growth. Genes Dev..

[19] Solga AC (2015). RNA sequencing of tumor-associated microglia reveals Ccl5 as a stromal chemokine critical for neurofibromatosis-1 glioma growth. Neoplasia.

[20] Pong WW, Higer SB, Gianino SM, Emnett RJ, Gutmann DH (2013). Reduced microglial CX3CR1 expression delays neurofibromatosis-1 glioma formation. Ann. Neurol..

[21] Daginakatte GC, Gianino SM, Zhao NW, Parsadanian AS, Gutmann DH (2008). Increased c-Jun-NH2-kinase signaling in neurofibromatosis-1 heterozygous microglia drives microglia activation and promotes optic glioma proliferation. Cancer Res..

[22] Pan Y (2021). NF1 mutation drives neuronal activity-dependent initiation of optic glioma. Nature.

[23] Venkatesh HS (2015). Neuronal activity promotes glioma growth through neuroligin-3 secretion. Cell.

[24] Venkatesh HS (2017). Targeting neuronal activity-regulated neuroligin-3 dependency in high-grade glioma. Nature.

[25] Venkataramani V (2019). Glutamatergic synaptic input to glioma cells drives brain tumour progression. Nature.

[26] Venkatesh HS (2019). Electrical and synaptic integration of glioma into neural circuits. Nature.

[27] Buckingham SC (2011). Glutamate release by primary brain tumors induces epileptic activity. Nat. Med..

[28] Campbell SL, Buckingham SC, Sontheimer H (2012). Human glioma cells induce hyperexcitability in cortical networks. Epilepsia.

[29] Campbell SL (2015). GABAergic disinhibition and impaired KCC2 cotransporter activity underlie tumor-associated epilepsy. Glia.

[30] John Lin CC (2017). Identification of diverse astrocyte populations and their malignant analogs. Nat. Neurosci..

[31] Yu K (2020). PIK3CA variants selectively initiate brain hyperactivity during gliomagenesis. Nature.

[32] Jett K, Friedman JM (2010). Clinical and genetic aspects of neurofibromatosis 1. Genet Med..

[33] Evans DG (2010). Birth incidence and prevalence of tumor-prone syndromes: estimates from a UK family genetic register service. Am. J. Med. Genet. A.

[34] Friedman JM (1999). Epidemiology of neurofibromatosis type 1. Am. J. Med. Genet..

[35] Li K (2016). Mice with missense and nonsense NF1 mutations display divergent phenotypes compared with human neurofibromatosis type I. Dis. Model Mech..

[36] Toonen JA (2016). NF1 germline mutation differentially dictates optic glioma formation and growth in neurofibromatosis-1. Hum. Mol. Genet..

[37] Omrani A (2015). HCN channels are a novel therapeutic target for cognitive dysfunction in Neurofibromatosis type 1. Mol. Psychiatry.

[38] Herrmann S, Schnorr S, Ludwig A (2015). HCN channels-modulators of cardiac and neuronal excitability. Int J. Mol. Sci..

[39] Santoro B, Shah MM (2020). Hyperpolarization-activated cyclic nucleotide-gated channels as drug targets for neurological disorders. Annu Rev. Pharm. Toxicol..

[40] Pinna V (2015). p.Arg1809Cys substitution in neurofibromin is associated with a distinctive NF1 phenotype without neurofibromas. Eur. J. Hum. Genet..

[41] Brannan CI (1994). Targeted disruption of the neurofibromatosis type-1 gene leads to developmental abnormalities in heart and various neural crest-derived tissues. Genes Dev..

[42] Jacks T (1994). Tumour predisposition in mice heterozygous for a targeted mutation in Nf1. Nat. Genet..

[43] Morcos P, Thapar N, Tusneem N, Stacey D, Tamanoi F (1996). Identification of neurofibromin mutants that exhibit allele specificity or increased Ras affinity resulting in suppression of activated ras alleles. Mol. Cell Biol..

[44] Koczkowska M (2018). Genotype-phenotype correlation in NF1: evidence for a more severe phenotype associated with missense mutations affecting NF1 codons 844-848. Am. J. Hum. Genet..

[45] Rojnueangnit K (2015). High incidence of noonan syndrome features including short stature and pulmonic stenosis in patients carrying NF1 missense mutations affecting p.Arg1809: genotype-phenotype correlation. Hum. Mutat..

[46] Bajenaru ML (2003). Optic nerve glioma in mice requires astrocyte Nf1 gene inactivation and Nf1 brain heterozygosity. Cancer Res..

[47] Anastasaki C (2020). Human iPSC-derived neurons and cerebral organoids establish differential effects of germline NF1 gene mutations. Stem Cell Rep..

[48] Le LQ, Shipman T, Burns DK, Parada LF (2009). Cell of origin and microenvironment contribution for NF1-associated dermal neurofibromas. Cell Stem Cell.

[49] Chen Z (2014). Cells of origin in the embryonic nerve roots for NF1-associated plexiform neurofibroma. Cancer Cell.

[50] Chen Z (2019). Spatiotemporal loss of NF1 in Schwann cell lineage leads to different types of cutaneous neurofibroma susceptible to modification by the Hippo pathway. Cancer Discov..

[51] Brossier NM, Thondapu S, Cobb OM, Dahiya S, Gutmann DH (2021). Temporal, spatial, and genetic constraints contribute to the patterning and penetrance of murine neurofibromatosis-1 optic glioma. Neuro Oncol..

[52] Lee DY, Gianino SM, Gutmann DH (2012). Innate neural stem cell heterogeneity determines the patterning of glioma formation in children. Cancer Cell.

[53] Mo, J. et al. Humanized neurofibroma model from induced pluripotent stem cells delineates tumor pathogenesis and developmental origins. *J. Clin. Investig.* **131**, 10.1172/JCI139807 (2021). 10.1172/JCI139807 PMC7773354 33108355

[54] Rodriguez FJ, Folpe AL, Giannini C, Perry A (2012). Pathology of peripheral nerve sheath tumors: diagnostic overview and update on selected diagnostic problems. Acta Neuropathol..

[55] Peltonen J, Penttinen R, Larjava H, Aho HJ (1986). Collagens in neurofibromas and neurofibroma cell cultures. Ann. N. Y Acad. Sci..

[56] Keilhoff G, Stang F, Wolf G, Fansa H (2003). Bio-compatibility of type I/III collagen matrix for peripheral nerve reconstruction. Biomaterials.

[57] Chi H, Horie H, Hikawa N, Takenaka T (1993). Isolation and age-related characterization of mouse Schwann cells from dorsal root ganglion explants in type I collagen gels. J. Neurosci. Res..

[58] Kitano Y, Okamoto E, Saito K, Okano Y (1992). Effects of several growth factors on cultured neurofibroma cells. J. Dermatol Sci..

[59] Widemann BC (2014). Phase II trial of pirfenidone in children and young adults with neurofibromatosis type 1 and progressive plexiform neurofibromas. Pediatr. Blood Cancer.

[60] Brosseau JP (2021). Human cutaneous neurofibroma matrisome revealed by single-cell RNA sequencing. Acta Neuropathol. Commun..

[61] Anastasaki C, Woo AS, Messiaen LM, Gutmann DH (2015). Elucidating the impact of neurofibromatosis-1 germline mutations on neurofibromin function and dopamine-based learning. Hum. Mol. Genet..

[62] Atit RP, Crowe MJ, Greenhalgh DG, Wenstrup RJ, Ratner N (1999). The Nf1 tumor suppressor regulates mouse skin wound healing, fibroblast proliferation, and collagen deposited by fibroblasts. J. Investig. Dermatol..

[63] Poolos NP, Bullis JB, Roth MK (2006). Modulation of h-channels in hippocampal pyramidal neurons by p38 mitogen-activated protein kinase. J. Neurosci..

[64] D’Angelo I, Welti S, Bonneau F, Scheffzek K (2006). A novel bipartite phospholipid-binding module in the neurofibromatosis type 1 protein. EMBO Rep..

[65] Sherekar M (2020). Biochemical and structural analyses reveal that the tumor suppressor neurofibromin (NF1) forms a high-affinity dimer. J. Biol. Chem..

[66] Deraredj Nadim W (2016). Physical interaction between neurofibromin and serotonin 5-HT6 receptor promotes receptor constitutive activity. Proc. Natl Acad. Sci. USA.

[67] Vallee B (2012). Nf1 RasGAP inhibition of LIMK2 mediates a new cross-talk between Ras and Rho pathways. PLoS ONE.

[68] Wang HF (2011). Valosin-containing protein and neurofibromin interact to regulate dendritic spine density. J. Clin. Investig..

[69] Wang Y, Nicol GD, Clapp DW, Hingtgen CM (2005). Sensory neurons from Nf1 haploinsufficient mice exhibit increased excitability. J. Neurophysiol..

[70] Muller F (2003). HCN channels are expressed differentially in retinal bipolar cells and concentrated at synaptic terminals. Eur. J. Neurosci..

[71] Moosmang S (2001). Cellular expression and functional characterization of four hyperpolarization-activated pacemaker channels in cardiac and neuronal tissues. Eur. J. Biochem..

[72] Cho HJ, Staikopoulos V, Furness JB, Jennings EA (2009). Inflammation-induced increase in hyperpolarization-activated, cyclic nucleotide-gated channel protein in trigeminal ganglion neurons and the effect of buprenorphine. Neuroscience.

[73] Vasilyev DV (2007). Direct inhibition of Ih by analgesic loperamide in rat DRG neurons. J. Neurophysiol..

[74] Poolos, N. P. Hyperpolarization-activated cyclic nucleotide-gated (HCN) ion channelopathy in epilepsy. 4th edition edn. in *Jasper’s Basic Mechanisms of the Epilepsies* (eds Noebels, J. L., Avoli, M., Rogawski, M. A., Olsen, R. W. & Delgado-Escueta, A. V.). (National Center for Biotechnology Information, 2012). 22787677

[75] Badalamente MA, Hurst LC (2007). Efficacy and safety of injectable mixed collagenase subtypes in the treatment of Dupuytren’s contracture. J. Hand Surg. Am..

[76] Kharouf Q, Pinares-Garcia P, Romanelli MN, Reid CA (2020). Testing broad-spectrum and isoform-preferring HCN channel blockers for anticonvulsant properties in mice. Epilepsy Res..

[77] Riccioni G (2010). Ivabradine: from molecular basis to clinical effectiveness. Adv. Ther..

[78] Zhu Y (2001). Ablation of NF1 function in neurons induces abnormal development of cerebral cortex and reactive gliosis in the brain. Genes Dev..

[79] Bajenaru ML (2002). Astrocyte-specific inactivation of the neurofibromatosis 1 gene (NF1) is insufficient for astrocytoma formation. Mol. Cell Biol..

[80] Liao CP (2018). Contributions of inflammation and tumor microenvironment to neurofibroma tumorigenesis. J. Clin. Investig..

[81] Hegedus B (2008). Preclinical cancer therapy in a mouse model of neurofibromatosis-1 optic glioma. Cancer Res..

