# Supplementary material for: Neuronal hyperexcitability drives central and peripheral nervous system tumor progression in models of neurofibromatosis-1
Source: Nat Commun. 2022 May 19;13:2785. doi: 10.1038/s41467-022-30466-6 (PMC9120229; doi:10.1038/s41467-022-30466-6)
Supplement: Supplementary file 1 — Supplementary Information [file 41467_2022_30466_MOESM1_ESM.pdf]

Supplementary Data

Neuronal hyperexcitability drives central and peripheral nervous system tumor progression in models of Neurofibromatosis-1

Corina Anastasaki, Juan Mo, Ji-Kang Chen, Jit Chatterjee, Yuan Pan, Suzanne M. Scheaffer, Olivia Cobb, Michelle Monje, Lu Q. Le, David H. Gutmann

Figures

Supplementary Figure 1. Characterization of the Arg1809Cys *Nf1*-mutant mouse strain; relative to Figures 1-7

Supplementary Figure 2. Analysis of Arg1809Cys *Nf1*-mutant neuron signaling; relative to Figures 2-3

Supplementary Figure 3. *Nf1*-mutant hippocampal neuron midkine secretion is dependent on neuronal hyperexcitability; relative to Figures 2-3

Supplementary Figure 4. Genetic silencing of *Hcn1* and *Hcn2* results in neuronal death; relative to Figures 2-3, 5-7

Supplementary Figure 5. Human sh*NF1* Schwann cell and sensory neuron analysis; relative to Figure 5

Supplementary Figure 6. COL1A2 is uniquely expressed by *NF1*-mutant peripheral nervous system neurons; relative to Figures 5-7

Tables

Supplementary Table 1. Antibodies used.

Supplementary Table 2. Oligonucleotides used for quantitative real-time PCR.

Supplementary Table 3. Microarray datasets used for *COL1A2* analysis.

References

References, relevant to Supplementary Table 1.

A

	WT	5425C>T+/-	5425C>T-/-
	2	2	0
	0	2	0
	3	3	0
	1	4	0
	1	3	0
	3	6	0
Total	13	20	0

B

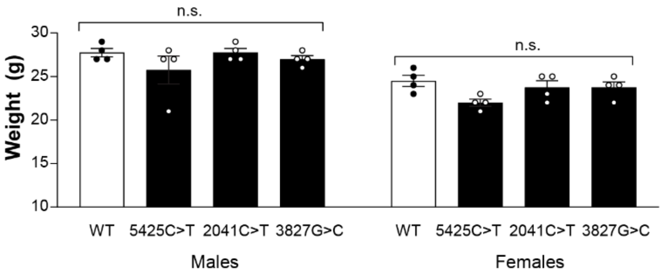

Supplementary Figure 1. Characterization of the Arg1809Cys *Nf1*-mutant mouse strain

A. Summary of litters of Arg1809Cys *Nf1* heterozygous mouse intercrosses.

B. Weights of male and female Arg1809Cys *Nf1*-mutant mice are similar to WT littermates at 1 month of age. n=4 for all groups.

Data are represented as means ± SEM. One-way ANOVA with Bonferroni post-test correction. p values were not significant (ns). Source data are provided as a Source Data file.

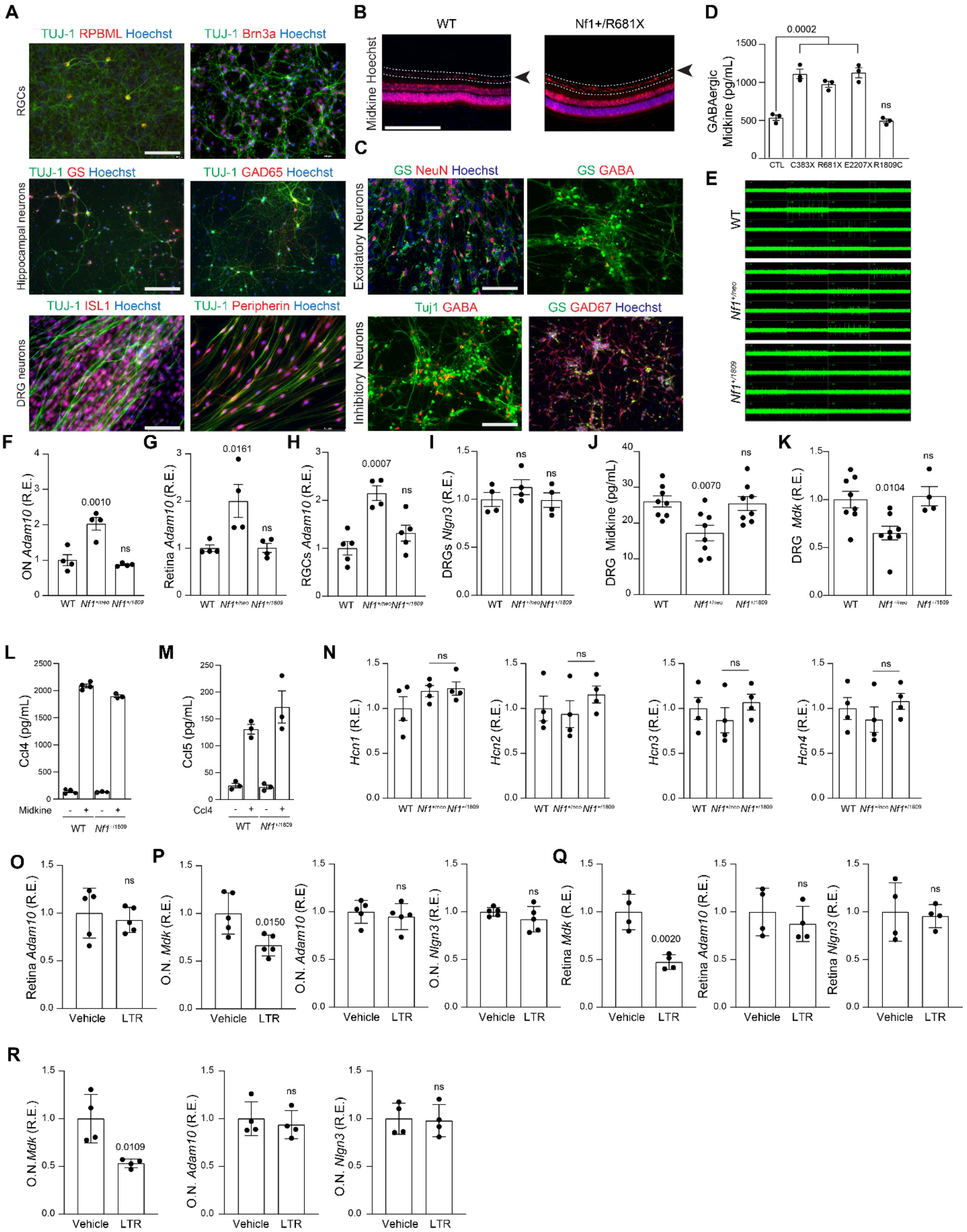
A
TUJ-1 RPBML Hoechst
TUJ-1 Brn3a Hoechst
RGCs
TUJ-1 GS Hoechst
TUJ-1 GAD65 Hoechst
Hippocampal neurons
TUJ-1 ISL1 Hoechst
TUJ-1 Peripherin Hoechst
DRG neurons
B
WT
Nf1+/R681X
Midkine Hoechst
C
GS NeuN Hoechst
GS GABA
Excitatory Neurons
Tuj1 GABA
GS GAD67 Hoechst
Inhibitory Neurons
D
0.0002
GABAergic Midkine (pg/mL)
ns
CTL
C383X
R681X
E2207X
R1809C
E
WT
Nf1+/neo
Nf1+/1809
F
ON Adam10 (R.E.)
0.0010
ns
WT
Nf1+/neo
Nf1+/1809
G
Retina Adam10 (R.E.)
0.0161
ns
H
RGCs Adam10 (R.E.)
0.0007
ns
I
DRGs Nlgn3 (R.E.)
ns
ns
J
DRG Midkine (pg/mL)
0.0070
ns
K
DRG Mdk (R.E.)
0.0104
ns
L
Ccl4 (pg/mL)
Midkine
WT
Nf1+/1809
M
Ccl5 (pg/mL)
Ccl4
WT
Nf1+/1809
N
Hcn1 (R.E.)
Hcn2 (R.E.)
Hcn3 (R.E.)
Hcn4 (R.E.)
ns
O
Retina Adam10 (R.E.)
ns
Vehicle
LTR
P
O.N. Mdk (R.E.)
0.0150
O.N. Adam10 (R.E.)
O.N. Nlgn3 (R.E.)
ns
Q
Retina Mdk (R.E.)
0.0020
Retina Adam10 (R.E.)
Retina Nlgn3 (R.E.)
ns
R
O.N.Mdk (R.E.)
0.0109
O.N. Adam10 (R.E.)
O.N. Nlgn3 (R.E.)
ns
Vehicle
LTR

Supplementary Figure 2. Analysis of Arg1809Cys *Nf1*-mutant neuron signaling

A. Immunocharacterization of retinal ganglion cells (RGCs; $RPBML^+$, $Brn3a^+$, $TUJ\text{-}1^+$), hippocampal neurons (Glutamate $Synthetase^+$, $GAD65^+$, $TUJ\text{-}1^+$) and dorsal root ganglia (DRG) neurons ($peripherin^+$, $Islet\text{-}1^+$, $TUJ\text{-}1^+$). Scale bar, 100 µm. Immunostaining of primary neurons was repeated independently 4 times with similar results.

B. Midkine expression is increased in $Nf1^{+/R681X}$ mutant RGCs relative to controls. Scale bar, 50 µm. Immunostaining of mouse retinae was repeated on a minimum of 3 independent animals per genotype with similar results.

C. Immunocharacterization of excitatory (Glutamate $Synthetase^+$, $NeuN^+$, $TUJ\text{-}1^+$) and inhibitory ($GABA^+$, $GAD67^+$, $TUJ\text{-}1^+$) hiPSC-derived CNS neurons. Scale bar, 100 µm. Immunostaining of hiPSC-derived neurons was repeated independently 3 times with similar results.

D. Midkine expression is increased in human CNS inhibitory (GABAergic) $NF1^{C383X}$, $NF1^{R681X}$ and $NF1^{E2207X}$, but not in $NF1^{R1809C}$, mutant neurons relative to controls (CTL). n=3 for all groups, p=0.0002.

E. Representative summary of multi-electrode array recordings of WT, $Nf1^{+/neo}$ and $Nf1^{+/1809}$ RGCs illustrating action potentials detected by each electrode over a period of 5 minutes.

F-H. Adam-10 (*Adam10*) transcript expression is increased in $Nf1^{+/neo}$ relative to WT and $Nf1^{+/1809}$ optic nerves, retinae and RGCs. **F**, n=4 for all groups, p=0.0010; **G,** n=4 for all groups, p=0.0161; **H**, WT n=5, $Nf1^{+/neo}$, $Nf1^{+/1809}$ n=4, p=0.0007

I. *Nlgn3* transcript expression is similar in $Nf1^{+/neo}$, $Nf1^{+/1809}$ and WT DRG neurons. n=4 for all groups.

J-K. Midkine *(Mdk)* (**J**) RNA and (**K**) protein expression are not increased in *Nf1*-mutant DRG neurons relative to WT controls. **J**, n=8 for all groups, p=0.007. **K**, WT n=8, $Nf1^{+/neo}$n=8, $Nf1^{+/1809}$ n=4, p=0.0104.

L. Ccl4 expression is increased similarly in WT (n=4) and Arg1809Cys *Nf1*-mutant (n=3) hippocampal neurons following midkine treatment.

M. Ccl5 is similarly elevated in WT (n=3) and Arg1809Cys *Nf1*-mutant (n=3) hippocampal neurons following Ccl4 treatment.

N. *Hcn1-4* RNA expression is not different in *Nf1*-mutant RGC neurons relative to WT controls. n=4 for all groups.

O. *Adam10* transcript expression is unchanged in retinae of 12 week-old *Nf1*$^{+/neo}$ mice following *in vivo* lamotrigine (LTR; 200µM) treatment. n=5 for all groups.

P. *Mdk, Adam10*, and *Nlgn3* transcript expression in the optic nerves (O.N.) of 12-week-old *Nf1*$^{+/neo}$ mice following *in vivo* LTR treatment. n=5 for all groups, O.N. *Mdk* R.E.p=0.015.

Q-R. *Mdk, Adam10,* and *Nlgn3* transcript expression in (**Q**) retinae (*Mdk* R.E. p=0.0020) and (**R**) optic nerves (*Mdk* R.E. p=0.0109) of 12 week-old *Nf1*-OPG mice following *in vivo* LTR treatment. n=4 for all groups.

Data are represented as means ± SEM. (**D, F-N**) One-way ANOVA with (**D, F-K**) Dunnett's or (**N**) Bonferroni post-test correction (**O-R**) two-tailed paired t-test. p values are indicated within each panel. ns, not significant. Source data are provided as a Source Data file.

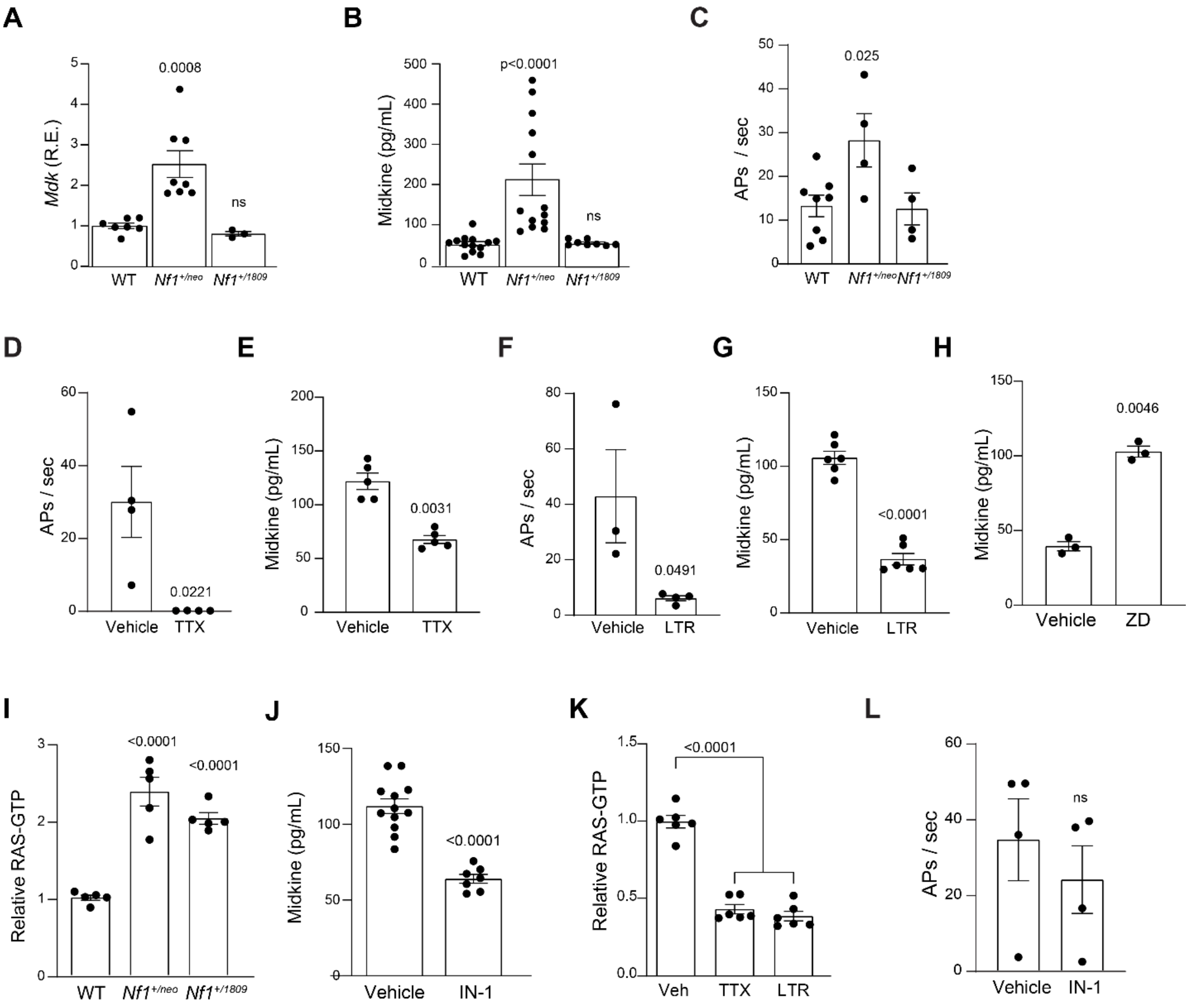

Supplementary Figure 3. ***Nf1*-mutant hippocampal neuron midkine secretion is dependent on neuronal hyperexcitability**

A-B. Midkine (**A**) transcript (*Mdk)* and (**B**) protein expression are increased in hippocampal neurons from *Nf1*$^{+/\text{neo}}$ mice (n=8; *Mdk* R.E. p=0.0008; Midkine p<0.0001) relative to WT controls (n=7) and *Nf1*$^{+/1809}$ mice (n=3).

C. Hippocampal neuron activity, as measured by action potential (AP) firing rates, is increased in *Nf1*$^{+/\text{neo}}$ (n=4; p=0.025) relative to WT (n=8) and *Nf1*$^{+/1809}$ (n=4) neurons.

D-G. (**D-E**) Tetrodotoxin (TTX; 1µM; **D**, n=4 for both groups, p=0.0221; **E**, n=5 for both groups, p=0.0002) and (**F-G**) lamotrigine (LTR; 200µM; **F,** vehicle n=3, LTR n=4, p=0.0491; **G,** n=6 for both groups, p<0.001) reduced (**D**) AP firing rates of and (**E**) midkine expression in *Nf1*$^{+/\mathrm{neo}}$ hippocampal neurons.

H. ZD-7288 (30µM) increases midkine secretion by *Nf1*$^{+/\mathrm{neo}}$ hippocampal neurons. n=3 for both groups, p=0.0046.

I. RAS activity is elevated in *Nf1*$^{+/neo}$ and *Nf1*$^{+/1809}$ hippocampal neurons relative to WT controls. n=5 for both groups, p<0.0001.

J. Hippocampal neuron midkine secretion is reduced following IN-1 treatment (1µM). Vehicle n=12, IN-1 n=7, p<0.0001.

K. RAS activity is reduced in *Nf1*$^{+/neo}$ neurons following TTX and LTR treatment. n=6 for all groups, p<0.0001.

L. *Nf1*$^{+/neo}$ hippocampal neuron AP firing rates are not reduced following IN-1 treatment. n=4 for both groups.

Data are represented as means ± SEM, (**A-C**; **I, K**) One-way ANOVA with Dunnett's post-test correction or (**D-H**, **J, L**) unpaired two-tailed student's t-test. p values are indicated within each panel. ns, not significant. Source data are provided as a Source Data file.

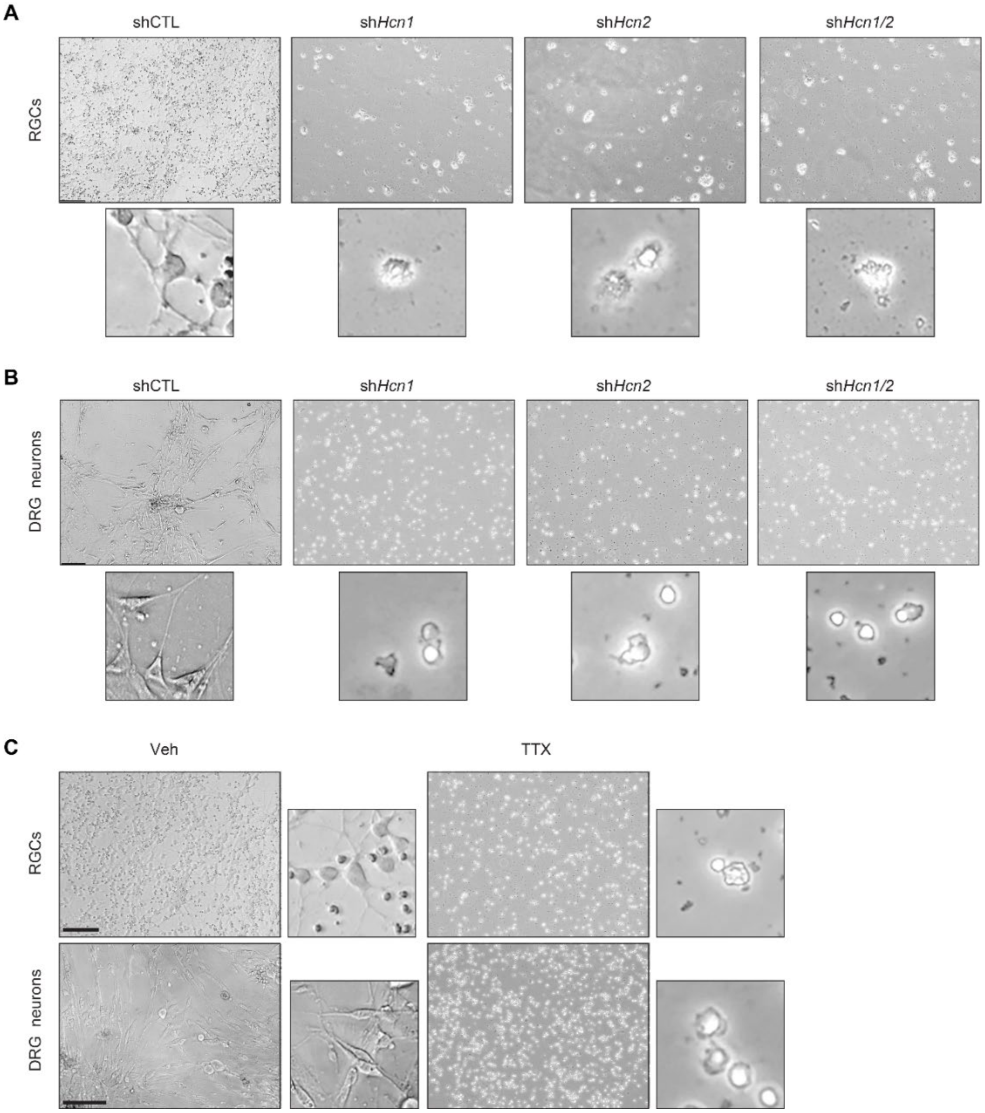

Supplementary Figure 4. Genetic silencing of *Hcn1* and *Hcn2* results in neuronal death.

A-B. Representative phase-contrast images depicting (**A**) RGCs or (**B**) DRG neurons infected with scrambled control, sh*Hcn1*, sh*Hcn2*, or a combination of sh*Hcn1* and sh*Hcn2*. Silencing of *Hcn1/2* lead to neuronal death. Independently generated primary RGC and DRG neurons were infected 3 times with similar results.

C. 6-hour treatment with TTX induced RGC and DRG neuronal cell death. Independently generated primary RGC and DRG neurons were treated with TTX 3 times with similar results.

Scale bars, 100µm

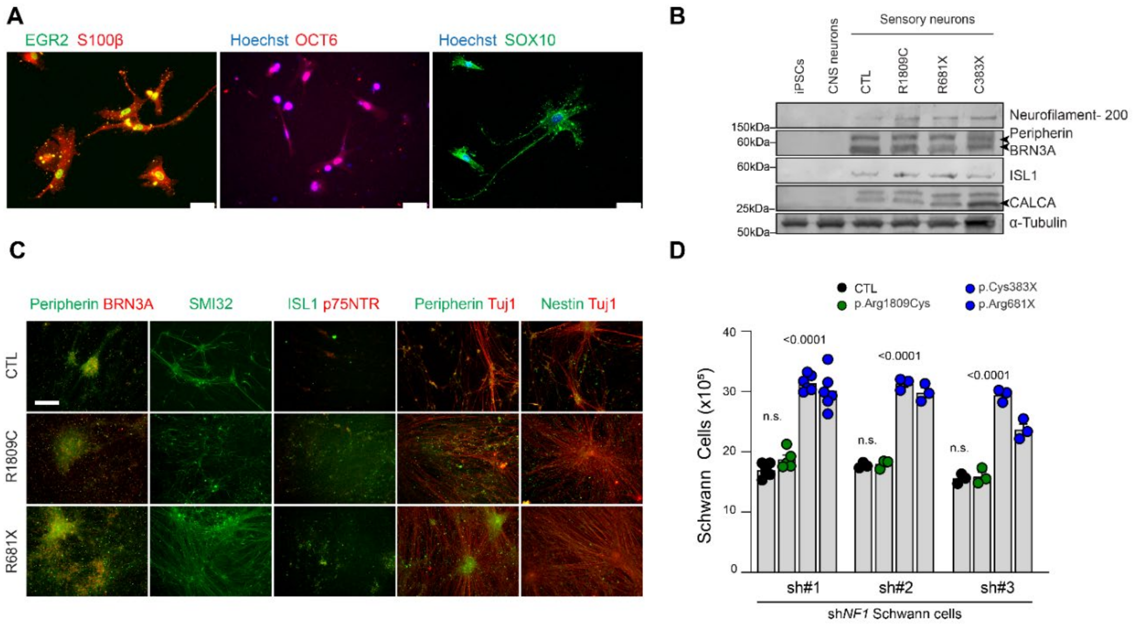

Supplementary Figure 5. Human sh*NF1* Schwann cell and sensory neuron analysis

A. Human sh*NF1* Schwann cells are immunopositive for EGR2, S100β, OCT6 and SOX10 expression. Immunostaining of human Schwann cells was repeated independently 3 times with similar results. Scale bars, 50 µm.

B-C. *NF1*$^{+/-}$ hiPSC-sensory neurons are immunopositive for (**B**) neurofilament, peripherin, BRN3A, ISL-1, and CALCA1 expression by western blot, as well as for (**C**) SMI32 and Tuj-1 by immunocytochemistry, but are immunonegative for Nestin and p75NTR expression. Scale bars, 50 µm. Immunostaining of hiPSC-sensory neurons was repeated independently a minimum of 3 times with similar results.

D. sh*NF1* human Schwann cell proliferation following hiPSC-sensory neuron CM treatment. CTL sh#1 n=6, Arg1809Cys sh#1 n=5, ns, Cys383X sh#1 n=6, p<0.0001, Arg681X sh#1 n=6, p<0.0001. CTL sh#2 n=3, Arg1809Cys sh#2 n=3, ns, Cys383X sh#2 n=3, p<0.0001, Arg681X sh#2 n=3, p<0.0001. CTL sh#3 n=3, Arg1809Cys sh#3 n=3, ns, Cys383X sh#3 n=3, p<0.0001, Arg681X sh#3 n=3, p<0.0001.

Data are represented as means ± SEM, 2-tailed paired t-tests or One-way ANOVA with Bonferroni post-test correction. p values are indicated within each panel. ns, not significant. Source data are provided as a Source Data file.

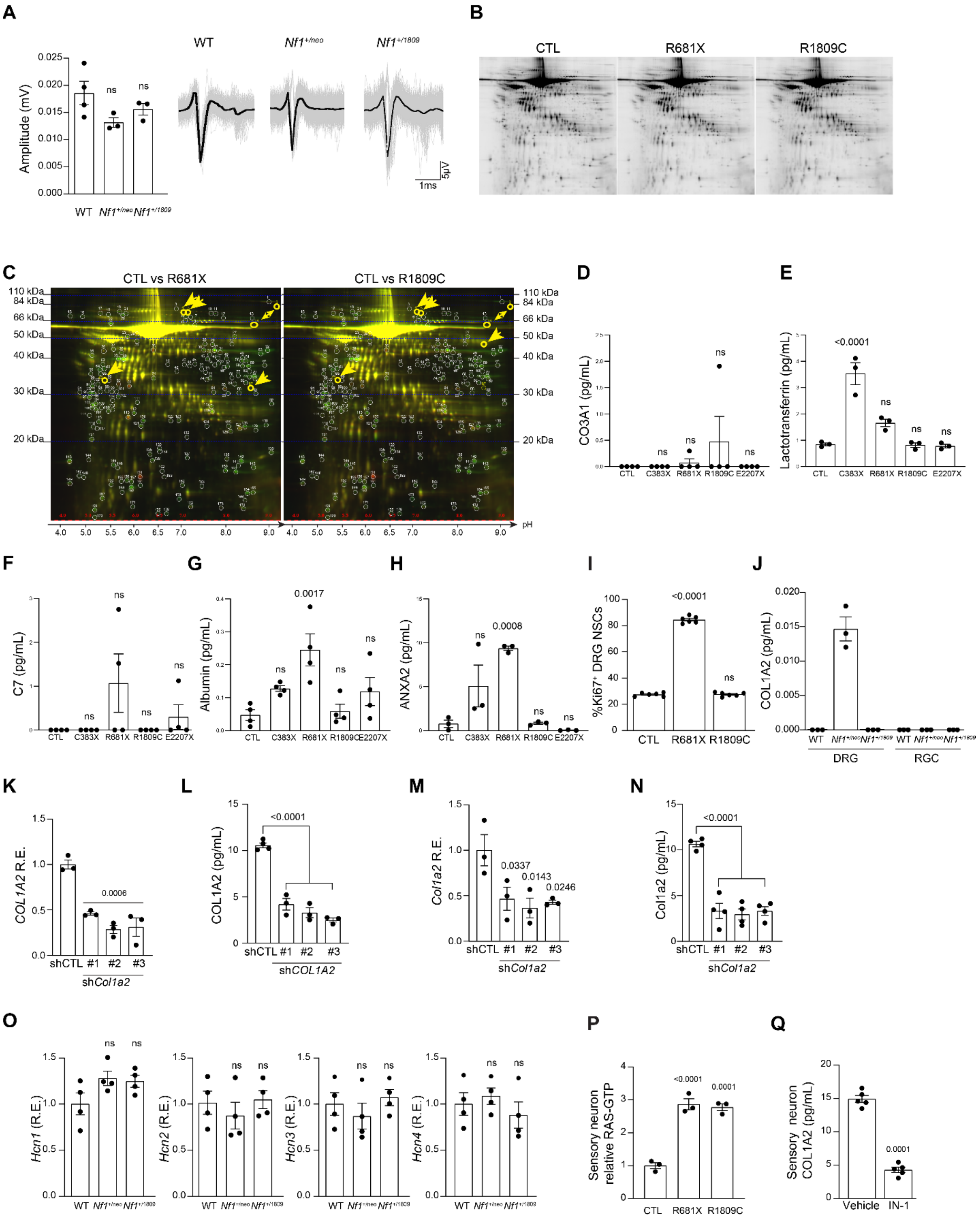
A
Amplitude (mV)
0.025
0.020
0.015
0.010
0.005
0.000
ns
ns
WT
Nf1+/neo
Nf1+/1809
5μV
1ms
B
CTL
R681X
R1809C
C
CTL vs R681X
CTL vs R1809C
110 kDa
84 kDa
66 kDa
50 kDa
40 kDa
30 kDa
20 kDa
4.0
5.0
5.5
6.0
6.5
7.0
8.0
9.0
pH
D
CO3A1 (pg/mL)
CTL
C383X
R681X
R1809C
E2207X
E
Lactotransferrin (pg/mL)
<0.0001
F
C7 (pg/mL)
G
Albumin (pg/mL)
0.0017
H
ANXA2 (pg/mL)
0.0008
I
%Ki67+ DRG NSCs
<0.0001
CTL
R681X
R1809C
J
COL1A2 (pg/mL)
DRG
RGC
K
COL1A2 R.E.
0.0006
shCTL
#1
#2
#3
shCol1a2
L
COL1A2 (pg/mL)
<0.0001
shCOL1A2
M
Col1a2 R.E.
0.0337
0.0143
0.0246
N
Col1a2 (pg/mL)
O
Hcn1 (R.E.)
Hcn2 (R.E.)
Hcn3 (R.E.)
Hcn4 (R.E.)
P
Sensory neuron relative RAS-GTP
0.0001
Q
Sensory neuron COL1A2 (pg/mL)
Vehicle
IN-1

Supplementary Figure 6. COL1A2 is uniquely expressed by *NF1*-mutant peripheral nervous system neurons.

A. The amplitudes of action potentials were similar in *Nf1*$^{+/neo}$ (n=3) and *Nf1*$^{+/1809}$ (n=3) DRG neurons relative to WT controls (n=4). Right panels: representative traces of DRG neuron action potentials over 3 msec (gray). The averages of the DRG action potential traces are indicated in black.

B-C. (B) 2D gels of control (CTL), *NF1*$^{\mathrm{R681X}}$ (R681X) and *NF1*^{R1809C} (1809) human sensory neuron conditioned media and (**C**) annotation of increased (green) and decreased (red) proteins in CM of R681X (left) or R1809C (right) *NF1*-mutant relative to control sensory neurons.

D-H. COL2A1, lactotransferrin, C7, albumin and ANXA2 expression in independently-generated hiPSC-sensory neuron CM were not uniquely elevated in *NF1*$^{\mathrm{C383X}}$- and *NF1*^{R681X}-mutant neurons relative to controls and *NF1*^{R1809C}-mutant neurons. **D**, n=4 all groups. **E,** n=3 all groups, C383X p<0.0001. **F**, n=4 all groups. **G**, n=4 all groups, R681X p<00001. **H,** n=3 all groups, R681X p=0.0008.

I. Increased proliferation (%Ki67^{+} cells) of mouse *Nf1*$^{-/-}$ DRG-NSCs following treatment with human *NF1*$^{\mathrm{R681X}}$-, but not CTL- and *NF1*^{R1809C}-mutant, hiPSC-sensory neuron CM. n=6 all groups, p<0.0001.

J. Col1a2 expression is increased in mouse *Nf1*$^{+/neo}$ DRG neurons, but not in mouse *Nf1*$^{+/neo}$ RGC neurons. n=3 all groups.

K-N Genetic inhibition of (**K-L**) human *COL1A2* or (**M-N**) mouse *Col1a2* with three independent short hairpin constructs reduces *COL1A2* and *Col1a2* (**K, M**) transcript and (**L, N**) protein expression relative to a control scrambled short hairpin (shCTL). **K,** n=3 all groups, p=0.0006. **L**, shCTL n=4, sh*COL1A2* #1-3 n=3; p<0.0001. **M,** n=3 all groups; *shCol1a2* #1 *p*=0.0337, *shCol1a2* #2 p=0.0143, *shCol1a2* #3 p=0.0246. **N,** n=4 all groups, p<0.0001.

O. *Hcn1-4* expression is not altered in *Nf1*-mutant DRG neurons relative to WT controls. n=4 all groups.

P. RAS activity is increased in human *NF1*^{R681X}- and *NF1*$^{\mathrm{R1809C}}$-mutant hiPSC-sensory neurons relative to controls. n=3 all groups, R681X p<0.0001, R1809C p=0.0001.

Q. COL1A2 is reduced in *NF1*^{681X}-mutant hiPSC-sensory neurons following IN-1 treatment. n=5 both groups, p<0.0001.

Data are represented as means ± SEM, (**A**, **D-P**) One-way ANOVA with Dunnett's post-test correction or (**Q**) two-tailed paired t-test. p values are indicated within each panel. ns, not significant. Source data are provided as a Source Data file.

Supplementary Table 1. Antibodies used

Antibody	Dilution	Validation (application)	Product Link	Manufacturer	Catalog# Number
Alexa Fluor 488 goat anti-mouse secondary antibody	1:200	Fisher Scientific (IF/ICC)	https://www.thermofisher.com/antibody/product/Goat-anti-Mouse-IgG-H-L-Highly-Cross-Adsorbed-Secondary-Antibody-Polyclonal/A-11029	Fisher Scientific	A11029
Alexa Fluor 568 goat anti-rabbit secondary antibody	1:200	Fisher Scientific (IF/ICC)	https://www.thermofisher.com/antibody/product/Goat-anti-Rabbit-IgG-H-L-Cross-Adsorbed-Secondary-Antibody-Polyclonal/A-11011	Fisher Scientific	A11011
Anti-actin	1:5,000	CST (WB)	https://www.cellsignal.com/products/primary-antibodies/b-actin-13e5-rabbit-mab/4970	Cell Signaling Technologies	4970
Anti-alpha-tubulin	1:5,000	Sigma-Aldrich (WB)	https://www.sigmaaldrich.com/US/en/product/sigma/t9026	Sigma Aldrich	T9026
Anti-beta III Tubulin antibody [2G10]	1:1,000	Abcam (WB, ICC)	https://www.abcam.com/beta-iii-tubulin-antibody-2g10-neuronal-marker-ab78078.html	Abcam	ab78078
Anti-BRN3A, Rabbit monoclonal [EPR23257-285]	1:1,000	Abcam (IHC, WB)	https://www.abcam.com/brn3a-antibody-epr23257-285-ab245230.html	Abcam	ab245230
Anti-CD3 antibody	1:50	Abcam (IHC)	https://www.abcam.com/cd3-antibody-cd3-12-ab11089.html	Abcam	ab11089
Anti-CD34	1:2,500	Abcam (IHC)	https://www.abcam.com/cd34-antibody-ep373y-ab81289.html	Abcam	ab81289
Anti-CGRP (CALCA)	1:500	Abcam (WB)	https://www.abcam.com/cgrp-antibody-ab189786.html	Abcam	ab189786
Anti-Col1a2 (for mouse samples)	1:200	ThermoFisher (IHC)	https://www.thermofisher.com/antibody/product/COL1A2-Antibody-Polyclonal/PA5-106555	ThermoFisher	PA5-106555
Anti-COL1A2 antibody (for human samples)	1:200	Abcam (ICC)	https://www.abcam.com/col1a2-antibody-ab96723.html	Abcam	ab96723
Anti-EGR2	1:100	Abcam (ICC)	https://www.abcam.com/egr2-antibody-oti1f10-ab156765.html	Abcam	ab156765
Anti-Factor XIIIa	1:100	Abcam (IHC)	https://www.abcam.com/factor-xiiia-antibody-ac-1a1-ab1834.html	Abcam	ab1834

Anti-GABA	1:1,000	Sigma (ICC)	https://www.sigmaaldrich.com/US/en/product/sigma/a2052	Sigma Aldrich	A2052
Anti-GAD2, Rabbit monoclonal [D5G2]	1:500	CST (IF)	https://www.cellsignal.com/products/primary-antibodies/gad2-d5g2-xp-rabbit-mab/5843	Cell Signaling Technologies	5843S
Anti-GAP43	1:2000	Abcam (IHC)	https://www.abcam.com/gap43-antibody-neuronal-marker-ab12274.html	Abcam	ab12274
Anti-GFAP, Rat monoclonal [2.2B10]	1:500	ThermoFisher (IHC, ICC, IF)	https://www.thermofisher.com/antibody/product/13-0300.html?ef_id=EAIaIQobChMIlby2rZKR9wIV7m1vBB3ySAD5EAAYASAAEgKDafD_BwE:G:s&s_kwcid=AL!3652!3!459736943987!!!g!!&cid=bid_pca_aup_r01_co_cp1359_pjt0000_bid00000_0se_gaw_dy_pur_con&gclid=EAIaIQobChMIlby2rZKR9wIV7m1vBB3ySAD5EAAYASAAEgKDafD_BwE	ThermoFisher	13-0300
Anti-Glutamate synthetase mouse monoclonal [3B6]	1:500	Abcam (ICC)	https://www.abcam.com/glutamine-synthetase-antibody-3b6-bsa-and-azide-free-ab64613.html	Abcam	ab64613
Anti-Glutamate synthetase rabbit polyclonal	1:500	Abcam (ICC)	https://www.abcam.com/glutamine-synthetase-antibody-ab228590.html	Abcam	ab228590
Anti-Iba1	1:500	Wako (IHC)	https://labchem-wako.fujifilm.com/us/category/01213.html	Wako	NC9288364
Anti-ISL-1 Rabbit monoclonal [EP4182]	1:250	Abcam (ICC)	https://www.abcam.com/islet-1-antibody-ep4182-neural-stem-cell-marker-ab109517.html	Abcam	ab109517
anti-Ki-67, Mouse monoclonal [B56]	1:500	BD Biosciences (ICC), (1)	https://www.fishersci.com/shop/products/anti-ki-67-clone-b56-bd-6/BDB556003	BD Biosciences	BDB556003
Anti-Midkine C-terminal	1:200	Abcam (IHC/IF), (2)	https://www.abcam.com/midkine-antibody-c-terminal-ab170820.html	Abcam	ab170820

Anti-Mouse IgG Polyclonal Secondary Antibody IRDye® 800RD	1:5,000	Li-Cor Biosciences (WB)	https://www.licor.com/bio/reagents/irdye-800cw-goat-anti-mouse-igg-secondary-antibody?ppc_keyword=&gclid=EAIaIQobChMI8fCg25OR9wIVNntvBB1Y-AkcEAAYASAAEgK8mPD_BwE	Li-Cor Biosciences	926-32210
Anti-Nestin	1:200	Abcam (ICC)	https://www.abcam.com/nestin-antibody-neural-stem-cell-marker-ab92391.html	Abcam	ab92391
Anti-NeuN mouse monoclonal [A60]	1:500	Sigma-Aldrich (ICC)	https://www.sigmaaldrich.com/US/en/product/mm/mab377	Sigma-Aldrich	MAB377
anti-Neurofilament H (NF-H), Nonphosphorylated Antibody mouse monoclonal SMI32	1:500	Biolegend (WB, IF)	https://www.biolegend.com/en-us/products/purified-anti-neurofilament-h-nf-h-nonphosphorylated-antibody-11475	Biolegend	801701
Anti-neuroligin-3	0.4µg/mL	Novus (WB), (1)	https://www.novusbio.com/products/neuroligin-3-nlgn3-antibody_nbp1-90080	Novus	NBP1-90080
Anti-OCT6	1:200	Abcam (ICC)	https://www.abcam.com/oct6-antibody-ab272925.html	Abcam	ab272925
Anti-p75 NTR rabbit monoclonal [D4B3]	1:1000	CST (ICC)	https://www.cellsignal.com/products/primary-antibodies/p75ntr-d4b3-xp-rabbit-mab/8238	Cell Signaling Technologies	8238S
Anti-peripherin	1:500	Abcam (ICC)	https://www.abcam.com/peripherin-antibody-ab4666.html	Abcam	ab4666
Anti-Rabbit IgG Polyclonal Secondary Antibody IRDye® 680RD	1:5,000	Li-Cor Biosciences (WB)	https://www.licor.com/bio/reagents/irdye-680rd-goat-anti-rabbit-igg-secondary-antibody	Li-Cor Biosciences	926-68071
Anti-Rbpms	1:1,000	PhosphoSolutions (ICC/ IF)	https://www.phosphosolutions.com/products/anti-rbpms-antibody-1832-rbpms	PhosphoSolutions	1832-RBPMS
Anti-S100β antibody, Rabbit monoclonal [EP1576Y]	1:100	Abcam (ICC)	https://www.abcam.com/s100-beta-antibody-ep1576y-astrocyte-marker-ab52642.html	Abcam	ab52642

Anti-SOX10 antibody, Rabbit monoclonal [EPR4007-104] (for mouse tumors)	1:250	Abcam (IHC), (3)	https://www.abcam.com/sox10-antibody-epr4007-104-ab180862.html	Abcam	ab180862
Anti-SOX10, Rabbit monoclonal [SP267] (for human cells)	1:50	Abcam (ICC)	https://www.abcam.com/sox10-antibody-sp267-ab227680.html	Abcam	ab227680
Biotinylated anti Mouse secondary antibody	1:200	Fisher (IHC)	https://vectorlabs.com/products/antibodies/biotinylated-goat-anti-mouse-igg	Vector Laboratories	BA9200
Biotinylated anti Rabbit secondary antibody	1:200	Fisher (IHC)	https://vectorlabs.com/products/antibodies/biotinylated-goat-anti-rabbit-igg	Vector Laboratories	BA-1000

WB, Western blot; IHC, immunohistochemistry; IF, immunofluorescence; ICC, immunocyctochemistry

Supplementary Table 2. Oligonucleotides used for quantitative real-time PCR

Oligonucleotides	Manufacturer	Catalog Number
ADAM10 - TaqMan® Gene Expression Assay FAM-MGB	ThermoFisher	Hs01109562_m1
Adam10 - TaqMan® Gene Expression Assay FAM-MGB	ThermoFisher	Mm00545742_m1
COL1A2 - TaqMan® Gene Expression Assay FAM-MGB	ThermoFisher	Hs01028940_g1
Col1a2 - TaqMan® Gene Expression Assay FAM-MGB	ThermoFisher	Mm00483888_m1
GAPDH - TaqMan® Gene Expression Assay FAM-MGB	ThermoFisher	Hs02786624_g1
Gapdh - TaqMan® Gene Expression Assay FAM-MGB	ThermoFisher	Mm99999915_g1
Hcn1 - TaqMan® Gene Expression Assay FAM-MGB	ThermoFisher	Mm00468832_m1
Hcn2 - TaqMan® Gene Expression Assay FAM-MGB	ThermoFisher	Mm00468538_m1
Hcn3 - TaqMan® Gene Expression Assay FAM-MGB	ThermoFisher	Mm01212852_m1
Hcn4 - TaqMan® Gene Expression Assay FAM-MGB	ThermoFisher	Mm01176084_m1
Mdk - TaqMan® Gene Expression Assay FAM-MGB	ThermoFisher	Mm00440279_m1
NLGN3 - TaqMan® Gene Expression Assay FAM-MGB	ThermoFisher	Hs01043809_m1
Nlgn3 - TaqMan® Gene Expression Assay FAM-MGB	ThermoFisher	Mm01225951_m1

Supplementary Table 3. Available microarray datasets used for *COL1A2* analysis.

Sample	Sample name	GSM352---
normal SCs	batch1c-NHSC_303_HG_U133_Plus_2.CEL	487
normal SCs	batch1c-NHSC_339_HG_U133_Plus_2.CEL	489
normal SCs	batch1c-NHSC_771_HG_U133_Plus_2.CEL	490
normal SCs	batch2a-NHSC216_HG_U133_Plus_2.CEL	501
normal SCs	batch2b-NHSC323_HG_U133_Plus_2.CEL	515
normal SCs	batch2c-NHSC338_HG_U133_Plus_2.CEL	526
normal SCs	batch3a-NHSC_286_HG_U133_Plus_2.CEL	535
normal SCs	batch3b-NHSC_J017_HG_U133_Plus_2.CEL	543
normal SCs	batch3c-NHSC_02.8_HG_U13Plus_2.CEL	550
normal SCs	batch3c-NHSC_J037_HG_U13Plus_2.CEL	551
cNF	batch3a-dNFSC_ERS_HG_U133_Plus_2.CEL	534
cNF	batch3b-dNFSC_ABB_HG_U133_Plus_2.CEL	542
cNF	batch3c-dNFSC_JLM_HG_U13Plus_2.CEL	549
cNF	batch1c-ABC_8N_-l-_HG_U133_Plus_2.CEL	479
cNF	batch1c-AIBC_2N_-l-_HG_U133_Plus_2.CEL	480
cNF	batch1c-CLT_6N_+l-_HG_U133_Plus_2.CEL	481
cNF	batch1c-MGF_33N_+l-_HG_U133_Plus_2.CEL	483
cNF	batch1c-SCC_7N_-l-_HG_U133_Plus_2.CEL	492
cNF	batch2a-ADN1N_KO_HG_U133_Plus_2.CEL	495
cNF	batch2b-RMN9N_KO_HG_U133_Plus_2.CEL	520
cNF	batch2c-SCC5N_KO_HG_U133_Plus_2.CEL	531
pNF	batch3a-pNFSC_04.7_HG_U133_Plus_2.CEL	537
pNF	batch3a-pNFSC_05.4_HG_U133_Plus_2.CEL	538
pNF	batch3b-pNFSC_00.13_HG_U133_Plus_2.CEL	544
pNF	batch3b-pNFSC_05.5_HG_U133_Plus_2.CEL	545
pNF	batch3c-pNFSC_97.9_HG_U13Plus_2.CEL	552
pNF	batch1b-pNF00.6_HG_U133_Plus_2.CEL	464
pNF	batch1b-pNF95.3_HG_U133_Plus_2.CEL	466
pNF	batch1b-pNF95.6_HG_U133_Plus_2.CEL	467
pNF	batch2a-pNF03.3_HG_U133_Plus_2.CEL	503
pNF	batch2b-pNF04.4_HG_U133_Plus_2.CEL	516
pNF	batch2c-pNF05.3_HG_U133_Plus_2.CEL	527

References

1. *Pan Y, Hysinger JD, Barron T, Schindler NF, Cobb O, Guo X, et al. NF1 mutation drives neuronal activity-dependent initiation of optic glioma. Nature. 2021;594(7862):277-82.*
2. *Guo X, Pan Y, Xiong M, Sanapala S, Anastasaki C, Cobb O, et al. Midkine activation of CD8(+) T cells establishes a neuron-immune-cancer axis responsible for low-grade glioma growth. Nat Commun. 2020;11(1):2177.*
3. *Mo J, Anastasaki C, Chen Z, Shipman T, Papke J, Yin K, et al. Humanized neurofibroma model from induced pluripotent stem cells delineates tumor pathogenesis and developmental origins. J Clin Invest. 2021;131(1).*
