# Supplementary material for: Neuronal hyperexcitability drives central and peripheral nervous system tumor progression in models of neurofibromatosis-1
Source: Nat Commun. 2022 May 19;13:2785. doi: 10.1038/s41467-022-30466-6 (PMC9120229; doi:10.1038/s41467-022-30466-6)
Supplement: Supplementary file 2 — Description of Additional Supplementary Files [file 41467_2022_30466_MOESM2_ESM.pdf]

Title: Supplementary Movie 1.
Description: Representative video of WT RGC calcium imaging recordings.

Title: Supplementary Movie 2.
Description: Representative video of Nf1+/neo RGC calcium imaging recordings.

Title: Supplementary Movie 3.
Description: Representative video of Nf1+/1809 RGC calcium imaging recordings.

Title: Supplementary Movie 4.
Representative video of Nf1+/neo RGC calcium imaging recordings before TTX treatment.

Title: Supplementary Movie 5.
Description: Representative video of Nf1+/neo RGC calcium imaging recordings after TTX treatment.

Title: Supplementary Movie 6.
Representative video of Nf1+/neo RGC calcium imaging recordings before lamotrigine treatment.

Title: Supplementary Movie 7.
Description: Representative video of Nf1+/neo RGC calcium imaging recordings after lamotrigine treatment.

Title: Supplementary Movie 8.
Description: Representative video of Nf1+/neo RGC calcium imaging recordings before IN-1 treatment.

Title: Supplementary Movie 9.
Description: Representative video of Nf1+/neo RGC calcium imaging recordings after IN-1 treatment.

Title: Supplementary Movie 10.
Description: Representative video of WT DRG calcium imaging recordings.

Title: Supplementary Movie 11.
Description: Representative video of Nf1+/neo DRG calcium imaging recordings.

Title: Supplementary Movie 12.
Description: Representative video of Nf1+/1809 DRG calcium imaging recordings.

Title: Supplementary Movies 13.
Description: Representative video of Nf1+/neo DRG calcium imaging recordings before TTX and lamotrigine treatments.

Title: Supplementary Movie 14.
Description: Representative videos of Nf1+/neo DRG calcium imaging recordings after TTX treatment.

Title: Supplementary Movie 15.
Description: Representative videos of Nf1+/neo DRG calcium imaging recordings after lamotrigine treatment.

Title: Supplementary Movie 16.
Description: Representative video of Nf1+/neo DRG calcium imaging recordings before IN-1 treatment.

Title: Supplementary Movie 17.
Description: Representative video of Nf1+/neo DRG calcium imaging recordings after IN-1 treatment.
