# Supplementary material for: Neuronal hyperexcitability drives central and peripheral nervous system tumor progression in models of neurofibromatosis-1
Source: Nat Commun. 2022 May 19;13:2785. doi: 10.1038/s41467-022-30466-6 (PMC9120229; doi:10.1038/s41467-022-30466-6)
Supplement: Supplementary file 3 — Reporting Summary [file 41467_2022_30466_MOESM3_ESM.pdf]

nature portfolio

Corresponding author(s): David H Gutmann

Last updated by author(s): Apr 15, 2022

Reporting Summary

Nature Portfolio wishes to improve the reproducibility of the work that we publish. This form provides structure for consistency and transparency in reporting. For further information on Nature Portfolio policies, see our Editorial Policies and the Editorial Policy Checklist.

Statistics

For all statistical analyses, confirm that the following items are present in the figure legend, table legend, main text, or Methods section.

n/a	Confirmed	
☐	☒	The exact sample size (n) for each experimental group/condition, given as a discrete number and unit of measurement
☐	☒	A statement on whether measurements were taken from distinct samples or whether the same sample was measured repeatedly
☐	☒	The statistical test(s) used AND whether they are one- or two-sided<br>*Only common tests should be described solely by name; describe more complex techniques in the Methods section.*
☐	☒	A description of all covariates tested
☐	☒	A description of any assumptions or corrections, such as tests of normality and adjustment for multiple comparisons
☐	☒	A full description of the statistical parameters including central tendency (e.g. means) or other basic estimates (e.g. regression coefficient) AND variation (e.g. standard deviation) or associated estimates of uncertainty (e.g. confidence intervals)
☐	☒	For null hypothesis testing, the test statistic (e.g. F, t, r) with confidence intervals, effect sizes, degrees of freedom and P value noted<br>*Give P values as exact values whenever suitable.*
☒	☐	For Bayesian analysis, information on the choice of priors and Markov chain Monte Carlo settings
☒	☐	For hierarchical and complex designs, identification of the appropriate level for tests and full reporting of outcomes
☒	☐	Estimates of effect sizes (e.g. Cohen's d, Pearson's r), indicating how they were calculated

Our web collection on statistics for biologists contains articles on many of the points above.

Software and code

Policy information about availability of computer code

Data collection	Image Studio Lite Version 5.2 software and LAS AF Lite 3.2.0 were used to collect image data. Bio-Rad MPM6 v6.3 was used to collect colorimetric assay data and CFX Manager 3.1 software was used to collect and analyze qPCR data. MEA data were collected using Axion integrated studio (AxIS) version 2.5.1 software.
Data analysis	The analysis for this paper was generated using Partek Flow version 10.0 and STAR version 2.7.8a software for RNA databases ; GraphPad Prism (versions v5, v8.2.1, and v9.3.1) for statistical analyses , ImageJ (1.53a), Adobe Photoshop version 21.1.1. for Image editing, Image Studio Lite Version 5.2 software for western blot image editing; the Matlab programming environment to generate single neuron activity traces following calcium imaging; AXION Biosystems integrated studio (AxIS) version 2.5.1, the AXION Biosystems neural metric tool and Offline sorter x64 version 4 software were used for MEA analysis.

For manuscripts utilizing custom algorithms or software that are central to the research but not yet described in published literature, software must be made available to editors and reviewers. We strongly encourage code deposition in a community repository (e.g. GitHub). See the Nature Portfolio guidelines for submitting code & software for further information.

Data

Policy information about availability of data

All manuscripts must include a data availability statement. This statement should provide the following information, where applicable:

- Accession codes, unique identifiers, or web links for publicly available datasets
- A description of any restrictions on data availability
- For clinical datasets or third party data, please ensure that the statement adheres to our policy

All data supporting this study are available in the main article, Supplementary files and Source Data files available with this manuscript.

Field-specific reporting

Please select the one below that is the best fit for your research. If you are not sure, read the appropriate sections before making your selection.

☒ Life sciences ☐ Behavioural & social sciences ☐ Ecological, evolutionary & environmental sciences

For a reference copy of the document with all sections, see nature.com/documents/nr-reporting-summary-flat.pdf

Life sciences study design

All studies must disclose on these points even when the disclosure is negative.

Sample size	Sample sizes were chosen based on prior power calculations (80% confidence to detect 25% differences) and previously published experiments in our laboratory (Hegedus B, Cancer Research 2008; Kaul A, Neuro-Oncol 2015).
Data exclusions	No data was excluded
Replication	Multiple independently generated samples were used for all experiments. All attempts at replication were successful and are included in the data presented herein. The number of replicates is clearly indicated at the methods section as well as in each figure legend.
Randomization	The samples were randomly assigned to experimental groups.
Blinding	The investigators were blinded during data collection and analysis, whenever possible. Namely, collection of animals for tissue dissection, harvest or primary neuron culture could not be blinded, as each animal was requested by their unique animal ID. However, the investigators remained blinded to all subsequent molecular, histological and immunohistochemical analyses of animal tissues/ samples post-collection (tumor volume measurements, tumor immunostaining/ quantitation, transcript / protein expression). As primary neurons generated from a subset of mouse strains needed to undergo treatments, investigators could not be initially blinded to the genotypes of the primary cultured cells. Investigators remained blinded to all subsequent data analyses.

Reporting for specific materials, systems and methods

We require information from authors about some types of materials, experimental systems and methods used in many studies. Here, indicate whether each material, system or method listed is relevant to your study. If you are not sure if a list item applies to your research, read the appropriate section before selecting a response.

Materials & experimental systems

n/a	Involved in the study
☐	☒ Antibodies
☐	☒ Eukaryotic cell lines
☒	☐ Palaeontology and archaeology
☐	☒ Animals and other organisms
☒	☐ Human research participants
☒	☐ Clinical data
☒	☐ Dual use research of concern

Methods

n/a	Involved in the study
☒	☐ ChIP-seq
☒	☐ Flow cytometry
☒	☐ MRI-based neuroimaging

Antibodies

Antibodies used	Supplier names, catalog numbers, clone numbers, validated applications, links for the antibodies, and working concentrations are provided in Supplemental Table S1.
Validation	Each antibody used has been validated by the manufacturing company, in previously peer-reviewed articles, and was additionally completed for the relevant species and tissue in our laboratory following the manufacturer's instructions. We confirmed that each antibody used had the expected cellular localization and/or cell expression patterns by immunohistochemistry or expected molecular sizes by immunoblotting.

Eukaryotic cell lines

Policy information about cell lines

Cell line source(s)	Human iPSCs: Washington University School of Medicine, Genome Engineering and iPSCs Center; Normal human Schwann cells: ScienCell Research Laboratories.

Authentication	iPSCs were generated by the Genome Engineering and iPSCs Center core facility, sequenced, and validated by morphological and immunohistochemical methods (pluripotency markers). The commercially available Schwann cells were characterized by immunocytochemical methods.
Mycoplasma contamination	All cell lines were negative for mycoplasma contamination.
Commonly misidentified lines (See ICLAC register)	No commonly misidentified cell lines were used in the study.

Animals and other organisms

Policy information about studies involving animals; ARRIVE guidelines recommended for reporting animal research

Laboratory animals	Mus musculus mice were used for this study. Mice were maintained on a 12 light/ dark cycle in a barrier facility of 21C and 55% humidity, with ad libitum access to food and water. Heterozygous Nf1 c.5425C>T; Arg1809Cys mutant mice were generated by CRISPR/ Cas9 engineering directly into C57Bl/6J embryos, resulting in mice with one wild-type Nf1 allele and one missense R1809C mutation. The mutation was confirmed by directed sequencing (IDT Technologies). R1809C Nf1-mutant mice, as well as heterozygous R681X and c.3827G>C Nf1-mutant mice were backcrossed to C57Bl/6J and wild-type littermates were used as controls. For retinal analyses, control and Nf1-mutant mice were collected at 1 month of age. For primary neuronal cultures, mice were collected at 4-10 days old. For pNF studies, mice were generated with the R1809C mutation or a neomycin cassette inserted in exon 31 as the germline Nf1 allele and somatic Nf1 inactivation in Hoxb7-Cre cells (Nf1flox/-;Hoxb7-Cre; Nf1flox/1809;Hoxb7-Cre). Additionally, conditional knockout Nf1flox/flox; Hoxb7-Cre mice were used. These mice were collected at 6 months for tumor assessment. Optic glioma-prone mice were generated with the R1809C mutation or a neomycin cassette inserted in exon 31 as the germline Nf1 allele and somatic Nf1 inactivation in neuroglial progenitor cells (Nf1f/1809; hGFAP-Cre or Nf1f/neo; hGFAP-Cre mice). Littermate Nf1flox/flox mice were used as controls. For these experiments, mice were collected at 3 months. For light/dark-rearing experiments, eight Nf1+/neo mice were reared in the dark for 4 weeks from 4 weeks of age. Eight littermate controls were reared in normal 12h light/dark cycles. Mice of both sexes were randomly assigned to all experimental groups without bias, and the investigators were blinded until final data analysis during all of the experiments. For in vivo lamotrigine treatment of NF1-pNFs, 8 week-old athymic nude mice (4 males: 4 females; Charles River, Stock No 490) underwent surgery to implant pNF progenitor cells.
Wild animals	The study did not involve wild animals
Field-collected samples	The study did not involve field-collected samples
Ethics oversight	All experiments were performed under active Animal Studies Committee protocols at Washington University School of Medicine and UT Southwestern.

Note that full information on the approval of the study protocol must also be provided in the manuscript.
